# Larger workers outperform smaller workers across resource environments: An evaluation of demographic data using functional linear models

**DOI:** 10.1002/ece3.7239

**Published:** 2021-02-11

**Authors:** Natalie Z. Kerr, Rosemary L. Malfi, Neal M. Williams, Elizabeth E. Crone

**Affiliations:** ^1^ Department of Biology Tufts University Medford MA USA; ^2^ Department of Biology Duke University Durham NC USA; ^3^ Department of Biology University of Massachusetts‐Amherst Amherst MA USA; ^4^ Department of Entomology and Nematology University of California Davis CA USA

**Keywords:** *Bombus vosnesenskii*, callow size, colony age, development, egg production, functional linear models, larval survival

## Abstract

1. Behavior and organization of social groups is thought to be vital to the functioning of societies, yet the contributions of various roles within social groups toward population growth and dynamics have been difficult to quantify. A common approach to quantifying these role‐based contributions is evaluating the number of individuals conducting certain roles, which ignores how behavior might scale up to effects at the population‐level. Manipulative experiments are another common approach to determine population‐level effects, but they often ignore potential feedbacks associated with these various roles.
2. Here, we evaluate the effects of worker size distribution in bumblebee colonies on worker production in 24 observational colonies across three environments, using functional linear models. Functional linear models are an underused correlative technique that has been used to assess lag effects of environmental drivers on plant performance. We demonstrate potential applications of this technique for exploring high‐dimensional ecological systems, such as the contributions of individuals with different traits to colony dynamics.
3. We found that more larger workers had mostly positive effects and more smaller workers had negative effects on worker production. Most of these effects were only detected under low or fluctuating resource environments suggesting that the advantage of colonies with larger‐bodied workers becomes more apparent under stressful conditions.
4. We also demonstrate the wider ecological application of functional linear models. We highlight the advantages and limitations when considering these models, and how they are a valuable complement to many of these performance‐based and manipulative experiments.

## Graphical abstract

Functional linear models are an under‐utilized correlative statistical method that uses observational data to explore high dimensional ecological systems. Here, we evaluated how the number of workers of different sizes affect worker production in bumblebee colonies. Overall, we found larger workers outperform smaller workers in colonies across three resource environments

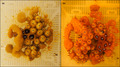

## 1 INTRODUCTION

In animal societies, individuals are often observed performing different tasks, such as guarding nests and burrows (Clutton‐Brock, Brotherton, et al., 2001), nursing, and caring for young (Kerth, 2008; Sparkman et al., 2011; Wilkinson, 1992), or reproducing (Faulkes & Bennett, 2001; Jarvis, 1981). The roles within these social groups are commonly assigned based on the age (Brent et al., 2015; Jarvis, 1981; Seeley & Kolmes, 1991; Zöttl et al., 2016), size (Goulson, 2009; Porter & Tschinkel, 1985; Schwander et al., 2005; Wenzel, 1992), and/or status (Frank, 1986; Sparkman et al., 2011) of individuals. For example, in Meerkats, which are cooperative breeders, younger nonbreeding individuals often stand on “sentinel duty” during group foraging bouts and care for offspring of the dominant breeding pair (Clutton‐Brock et al., 2004; Clutton‐Brock, Russell, et al., 2001; Clutton‐Brock et al., 2002). Without the co‐operation of these nonbreeders, the survival of individuals within the colonies is likely to decrease, particularly for the young (Doolan & Macdonald, 1999; Russell et al., 2007). This social behavior and organization is often assumed to be vital to the functioning and survival of these societies.

The most common approach to understanding the contribution of roles within social groups is to observe the behavior and performance of individuals. However, observing certain individuals performing a task does not mean they are better than other individuals at performing that task. To attempt to tackle the challenges associated with quantifying trait‐based contributions, a few studies have manipulated colonies in the laboratory to evaluate the effects of the social organization of age and size polymorphic species, such as mole rats (Jarvis, 1981; Zöttl et al., 2016), ants (Billick & Carter, 2007; Porter & Tschinkel, 1985), and bumblebees (Cnaani & Hefetz, 1994; Couvillon et al., 2010; Jandt & Dornhaus, 2009, 2011, 2014). In laboratory colonies of a eusocial ant *Pheidole dentata*, larvae gained more mass when reared by older workers, suggesting that older workers contribute more toward worker production in these ant colonies than their younger sisters (Muscedere et al., 2009). However, colonies within these laboratory experiments were not faced with the same external environmental stressors as those in the wild. In the case of bumblebees, larger workers are more susceptible to predators and parasites (Cartar & Dill, 1991; Malfi & Roulston, 2014; Muller et al., 1996), despite being better foragers. Therefore, the behaviors of social organism under artificial conditions might not capture all the feedbacks associated with size‐ or age‐based roles.

Functional linear models (FLMs) provide an additional method of inference about high‐dimensional ecological systems using observational data. For example, FLMs can evaluate the contributions of age‐ or size‐based roles within societies to population dynamics. These models assume that the effect of a predictor variable (e.g., number of workers) on a response variable (e.g., egg production) is a smooth function of some feature of the predictor variable (e.g., size of workers). Past applications of FLMs in ecology have investigated environmental drivers of plant population dynamics (Teller et al., 2016; Tenhumberg et al., 2018). These studies evaluated the effects of environmental conditions (e.g., precipitation) on plant performance (e.g., growth) assuming the slope of the effect of environmental conditions and plant performance varies as a smooth function of the time lag between conditions and performance (e.g., precipitation in the past 1, 2, 3… months). For example, the slope of precipitation versus plant growth could go from positive in recent months to zero at longer time lags. This method has potential for wider ecological application to investigate life‐history phenomena. Here, we explore application of FLMs to quantifying the relationship between aspects of new worker production as a function of the body size of existing workers in bumblebee colonies.

Bumblebees (*Bombus* spp.) are primitively eusocial insects that form relatively small colonies and have a discrete life cycle lasting only for a single season, which makes them a tractable system for studying trait‐based roles within societies. Bumblebees also exhibit worker size polymorphism, where workers within colonies vary up to 10‐fold in mass (Goulson, 2009). In bumblebee colonies, larger workers are often found foraging and guarding, while smaller workers spend more time in the colony conducting in‐nest tasks such as fanning and incubating (Cumber, 1949; Goulson et al., 2002; Inoue et al., 2010; Jandt & Dornhaus, 2009; Richards, 1946). Many studies have measured the importance of body size in determining how well workers perform various tasks, ranging from foraging and flight dynamics to thermoregulating and undertaking. Most of these have found that larger workers are better at multiple tasks, such as foraging and nursing (Cnaani & Hefetz, 1994; Goulson et al., 2002; Ings, 2007; Kerr et al., 2019; Peat & Goulson, 2005; Spaethe et al., 2007; Spaethe & Weidenmüller, 2002), with a few studies concluding either that intermediate size is better (Jandt & Dornhaus, 2014), or that there is no size‐based difference in performance (Jandt & Dornhaus, 2014). Although these studies demonstrate that body size affects worker performance at certain tasks, they do not demonstrate how their size‐based performance at tasks may, in turn, affect colony growth and development.

No studies have found smaller bumblebee workers to be better at performing tasks essential to colony function. However, smaller workers are more resilient to starvation (Couvillon & Dornhaus, 2010). Therefore, their value may become more apparent when food resources are limiting. In addition, smaller workers have lower production costs, so they may be more cost‐effective (Kerr et al., 2019). Here, we used FLMs to evaluate the contribution of workers of different sizes to worker production in bumblebee colonies under three different environments: a low‐resource environment; an environment with an early season pulse followed by low resources (“high‐low”); and a high‐resource environment. We looked at five vital rates relating to worker production: (a) number of new eggs laid, (b) development time, (c) larval survival, and (d) mean and (e) variance in worker emergence size, that is, the size of callow workers. By evaluating the contribution of different‐sized workers under different resources environments to worker production, we can assess whether larger workers are more beneficial when resource conditions are more favorable and whether the benefit of small workers to colonies is only seen when resources are low, making both production cost and resistance to starvation a premium.

## 2 MATERIALS AND METHODS

### 2.1 Study species and sites

We hand reared *Bombus vosnesenskii* colonies from wild‐caught queens collected at the University of California McLaughlin Reserve (N38 52 25.74, W122 25 56.25) in early spring 2015 and 2016 while they searched for nest sites. These colonies were the basis for two separate studies, both of which are previously published (Kerr et al., 2019; Malfi et al., 2019). Here, we use previously unpublished data (*Brood mapping,* below) from these studies to investigate effects of worker size on colony growth, so we briefly describe the rearing process.

In 2015 and 2016, we hand‐reared colonies in the laboratory in a dark room at 26–28°C for 6–9 weeks until their second or first cohort of worker bees eclosed. In 2015, we relocated seven colonies outside (N38 32 12.21, W121 47 16.95) at the Harry H. Laidlaw Jr. Honey Bee Research Facility (Davis, CA), where the surrounding landscape consisted of agricultural crops, floral research plots, and a 0.2‐ha pollinator garden (Figure S3a). In 2016, we relocated 14 colonies outside in agricultural fields at UC Davis Experimental Farm property (N38 31 32.3, W121 46 56.54). Half of the colonies (*n* = 7) had access to flight cages that provided a pulse of native California wildflower species for ~4 weeks early in the season (“pulse” treatment) and the other half had no supplemental forage (“control” treatment) (Malfi et al., 2019). The surrounding landscapes were croplands consisting of mainly nonflowering cereals, corn, and a strip of riparian habitat (Figure S3b).

In this study, we broadly categorized the resource environments experienced by our experimental colonies in each of these years based on observational differences in the quality and abundance of forage. The 2015 colonies, located next to a pollinator garden at the Honey Bee Research Facility, had the highest resource availability and quality (“high”), colonies in the 2016 pulse treatment had the second highest resource availability and quality (“high‐low”), and colonies in the 2016 control treatment had the lowest availability and quality (“low”). These three environments will now be referred to as high, high‐low, and low. Note that comparisons between the 2015 colonies and 2016 should be interpreted with the caveat that differences could be due to factors other than nutrition. Based on our observations, the most noticeable differences among treatments were the quality and abundance of floral resources (discussed further in the *Discussion*).

### 2.2 Brood mapping

**FIGURE 1:**
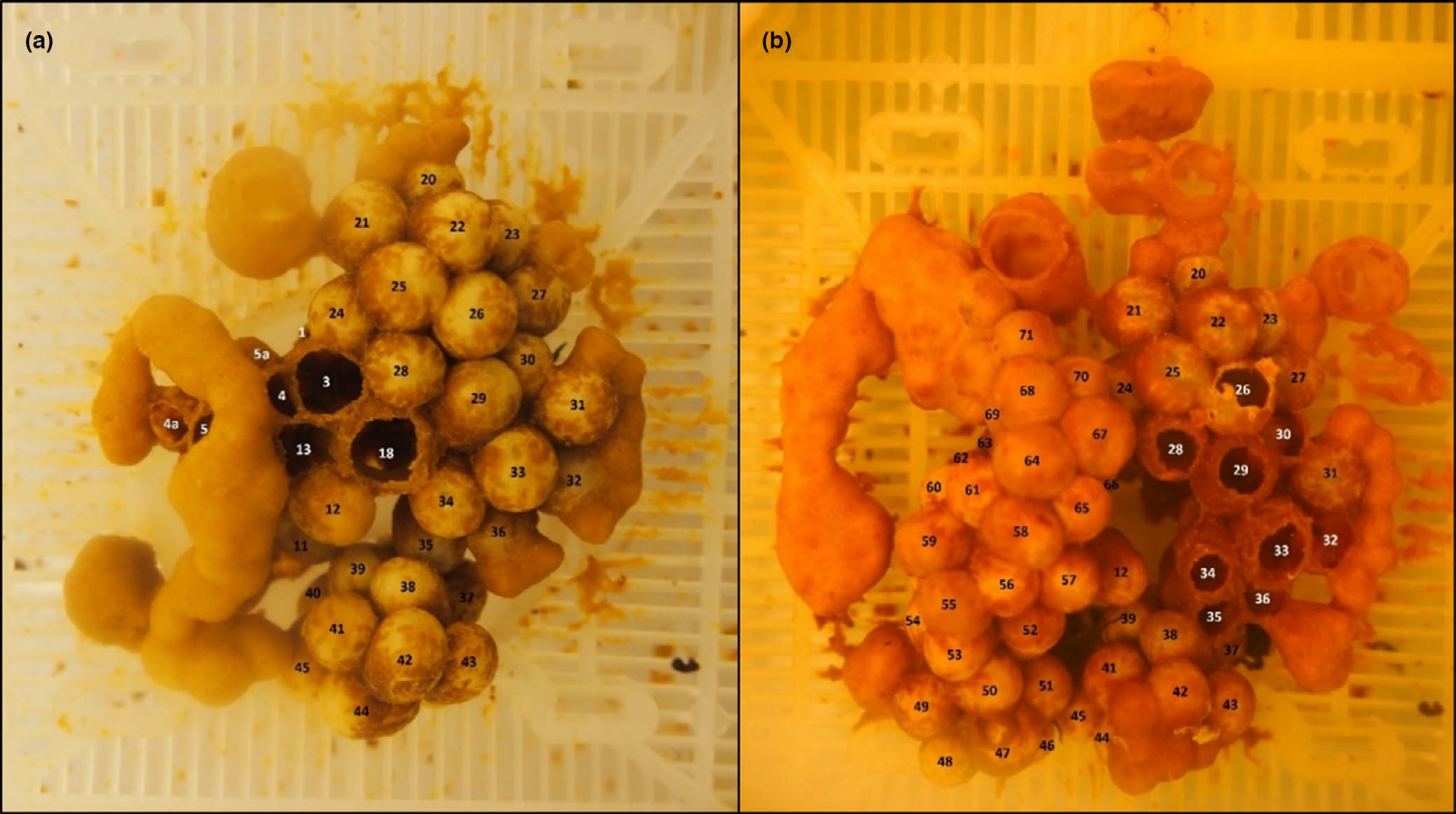
Example of brood mapping photographs used to track the fate of individual cells. These mapping photographs are aerial photographs for colony 6 in (a) week 5 and (b) week 6 since the first brood photograph. Aerial, side, and diagonal photographs were taken to capture all cells. Each cell has been individually numbered to track each cell. The larger stand‐alone open wax structures are honey pots

**FIGURE 2:**
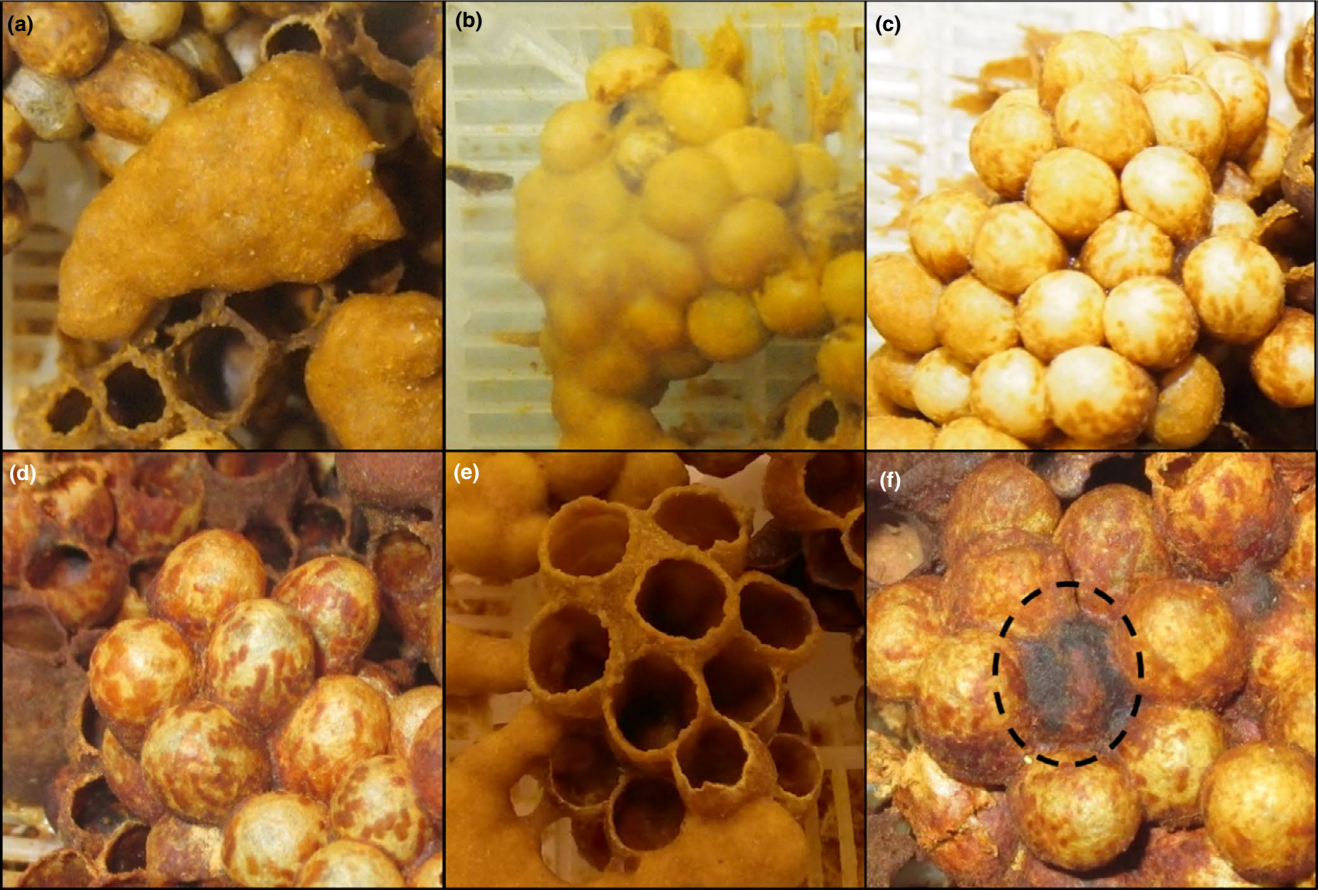
Brood mapping photos showing each of the six categories of living or dead stages of cell development. The six stages are: (a) clump stage, which are egg stages; (b) prepopcorn stages, which represents early larval instars; (c) popcorn stage, which are late instar larvae; (d) cocoon stage; (e) eclosed stage, and (f) a dead cell (dashed circle). These categories assisted with estimating three vital rates relating to worker production: eggs laid, development time, and larval survival

Each week, we photographed the brood from multiple angles (above, side, diagonal) to fully capture all brood cells. We individually numbered each brood cell in the photographs as it differentiated and tracked the fate of all marked cells throughout colony development (Figure 1). We classified each living brood cell into five categories: (a) clump stage, which represents the egg stage where individual cells have not yet differentiated; (b) predifferentiated stage, which represents early larval instars where individual cells have begun differentiating; (c) differentiated stage, which represents later larval instars where individual brood cells are clearly differentiated; (d) cocoon stage, where cells had darkened indicating that pupae have spun their cocoons; and (e) eclosed stage, where the cell has opened and an adult worker emerged (Figure 2 for stages). We also had two other categories: (f) dead, where we had observed a dead cell, and (g) unseen, where the cell could no longer be seen in the brood photograph.

Some brood clumps did not develop into distinct cells before the end of brood mapping, while other clumps died before cell partitioning. Rather than exclude these indistinct, dead, or undeveloped brood clumps in our analyses (*N*_low_ = 24/115; *N*_high‐low_ = 36/150; *N*_high_ = 36/163), which could result in underestimating egg production and overestimating larval survival, we estimated the number of cells for these clumps. We did this by classifying these indistinct brood clumps into five size categories (tiny, small, medium, large, and extra‐large) based on comparisons with similarly sized brood clumps that did divide into individual cells and assigning the mean value of cells for these size categories to indistinct clumps. From the 322 distinct clumps with a total of 3,917 cells with known fates, we estimated 432 cells from 96 indistinct clumps appeared to have died before differentiating, which comprises of less than 10% of total cells in our larval survival analyses.

From the brood mapping, we estimated three vital rates: egg production, larval development time, and larval survival. We considered weekly egg production to be the number of newly visible cells in either clump or predifferentiated stages. We assumed that the number of distinct cells formed by a brood clump represented the total number of eggs laid, that is, no eggs died before larval cells differentiated. We calculated development time for each cell as the number of days from when it was first seen as an egg (defined as the “clump” stage) to when it was first seen as an eclosed cell. Cells that were not detected in the clump stage or that disappeared from view before visibly eclosing were excluded from our analyses of larval development time. Finally, we classified larval survival as the success of each cell at surviving to eclosion. We excluded 43 unseen brood cells from our larval analyses because more than 8 days (50% the normal bumblebee development time) passed between photographs of them so their fates could not be unambiguously mapped. These represent 10% of 437 unseen cells or 1% of all 4,640 cells mapped across the 21 colonies and three resource environments.

### 2.3 Worker surveys

We conducted weekly night‐time surveys to estimate the mean and coefficient of variation (CV) in the size of newly emerged workers (hereafter referred to as “callow size”). We assigned each bee a unique tag using a combination enamel paint and numbered, color‐tags or Microsensys radio‐frequency identification (RFID) tags (Kerr et al., 2019; Malfi et al., 2019). For each newly emerged (“callow”) worker, we estimated body size by measuring intertegular (IT) span to the nearest 0.01 mm using digital calipers (Cane, 1987; Hagen & Dupont, 2013) and wet weight to the nearest 0.01 mg using an analytical microbalance (Mettler Toledo XS205DU). The size of each worker at initial capture was used to estimate the mean and CV of callow size. We used these size measurements in combination with presence/absence data to determine the number of workers of each size (now referred to as “worker size composition”) present in each colony for each week of the survey in order to evaluate the effects of worker size composition on aspects of worker production.

### 2.4 Functional linear models

**FIGURE 3:**
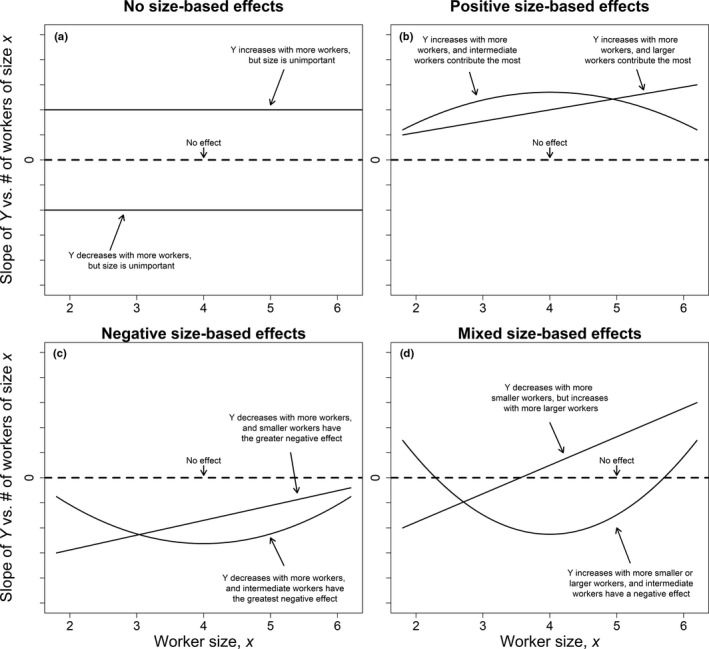
Example of functional linear model results showing the smooth function of the slopes of *Y* versus the number of workers as a function of worker size, *x*. *Y* covariate could be one of the five metrics of worker production: egg production, larval development time, larval survival, and mean and variance in callow size. We illustrate the following examples: (a) no size‐based per capita effect, but more workers of any size increases (*β*_0_ *> *0) or decreases (*β*_0_ < 0) *Y*; (b) positive size‐based per capita effects on *Y*; (c) negative size‐based per capita effects on *Y*; and (d) mixed size‐based per capita effects, that is, more workers of one size have negative effects and more workers of another size have positive effects. The dotted line on each panel represents no per capita effects of workers

We used functional linear models (FLMs) to estimate how five vital rates varied with worker size composition. FLMs are a type of regression spline that allows a covariate to vary smoothly over a continuous domain (Ramsay et al., 2009; Ramsay & Silverman, 2005). Therefore, instead of restricting our predictors (*X*) to unidimensional space (i.e., simple linear models, such as total worker number predicts number of eggs), we can evaluate the effect of the number of workers on some response variable (e.g., number of eggs) as a continuous function of worker size (i.e., a separate attribute of the predictor variable), such that the smooth function of size‐specific slopes versus worker size can be described as:

(1) $$\;E\left(Y\right)=\beta_{0}\;+\sum_{x=1}^{\max\left(x\right)}\left(s_{x}\right)W\left(n_{x}\right)$$

where $E\left(Y\right)$ is the expected value of the response variable *Y* (e.g., number of eggs); $\beta_{0}$ is the intercept; $W\left(n_{x}\right)\;$ is the number of workers *n* of size *x*; and β (*s*) is the slope of *Y* versus the number of workers of each size category *x* (c.f. methods in Teller et al., 2016). Here, the continuous attribute (i.e., worker size) of the predictor variable (i.e., number of workers) is discretized into many size categories (14 size categories for both low and high‐low, and 17 for high‐resource colonies) to approximate a continuous distribution of sizes (i.e., the worker size composition). The expected value of the response variable is the sum of the product of the size‐specific slopes β (*s_x_*) multiplied by the number of workers of size *x* (Figure 3). If the slope of *Y* versus the number of workers of size *x* is positive, then more workers of size *x* increase values of *Y* and vice versa when the slope is negative (Figure 3).

We parameterized the smooth functions of the size‐specific slopes using general additive models (GAMs). We fit our GAMs using the cubic spline basis for all smooth covariates, so that the coefficients will be set to 0 if our covariates have no effects on the response (see Zuur, 2012, for an excellent textbook introduction to GAMs). We used worker size composition in the previous week to predict both the number of eggs laid and larval survival in the present time step for our size composition FLMs. For the other three vital rates relating to worker production, we quantified worker size composition as the average number of workers in each size category across their larval development period.

Models were fit separately to data from each study (i.e., low‐, high‐low‐, and high‐resource environments), and we included colony ID as a fixed effect (i.e., a different intercept term for each colony) for each model to account for between‐colony effects. We used negative binomial GAMs to account for overdispersion for estimating new eggs laid and development time. We offset the number of new eggs laid by the number of days between brood photographs. We used binomial and Gaussian‐distributed GAMs for larval survival and callow size, respectively. We parameterized the binomial GAMs for estimating larval survival using successes and failures, where the total number of trials was defined as the number of days between brood photographs, and the number of successes was defined as the total number of days if the cell survived (i.e., zero failures) and the total number of days minus 1 if the cell died (i.e., one failure). We restricted the number of knots for our smooth terms of the number of workers of size *j* to a maximum of five. We also rejected any model structure that did not produce unimodal functions for our smooth term of worker size composition, since GAMs are prone to overfitting, and multimodal functions generally did not appear to be biologically meaningful. We used likelihood ratio tests to assess the fit of the parametric intercept term and the number of knots for each smooth term in our models given our data. We used cutoff of *p* < .05 for parametric terms and a cutoff of *p* < .01 for smooth terms, since *p* values for smooth terms are only approximate and are likely too low (Wood, 2017). We ran these general additive models (using mgcv::gam; Wood, 2004, 2011) in program R (R Core Team, 2017); see Appendix S1 for example code for our functional linear models.

To evaluate whether size‐specific slopes of worker size differed among treatments, we ran a model with all data combined and evaluated the AIC of the combined model with an AIC of models separated by treatment $({\text{AIC}}_{\mathrm{sep}}=\;2\times \left(k_{\mathrm{low}}+k_{high‐low}+k_{\mathrm{high}}\right)‐2\times \left(LL_{\mathrm{low}}+\;LL_{high‐low}+LL_{\mathrm{high}}\right)$ and by year (Table 1). We repeated all analyses with slopes scaled to size‐based worker production costs (see Appendix S2 for methods; Kerr et al., 2019 for production costs), rather than numbers of individuals. Because these results were largely parallel (Appendix S2), we do not discuss them further.

**TABLE 1:** dAIC values for functional linear models using data combined (i.e., no effect of treatment or year) for each daily vital rate

Vital rates	dAIC (models fit to all data)			dAIC (Pairwise comparisons)^1^		
	Combined	By treatment	By year	Low versus high‐low	Low versus high	High‐low versus high
Daily egg production	23.1	0	6.4	6.4	15.6	7.9
Development time (days)	352.7	0	48.2	5.2	96.8	272.3
Daily larval survival	24,004.1	23.2	0	−23.1	12,568.6	17,488.6
Mean callow size	10.8	3.1	0	−3.1	7.7	1.25
CV in callow size	41.4	0	11.5	11.5	34.2	40.8

^1^ AIC of models fit to data from both groups together, minus AIC of models fit to data from each treatment group separately. Positive values indicate significant differences between groups.

Colony size (i.e., number of observed workers) increased with colony age across three resource environments (Figure S2‐4). To avoid potentially confounding effects due to collinearity between colony age and worker number, we ran models separately with colony age and worker size composition as predictors of various measures of worker production success. Results for colony age are described in Appendix S3. Relationships between worker size composition and larval survival and mean callow size were somewhat confounded with colony age effects and should be interpreted with caution (Table 2, Appendix S4). We found no evidence for potentially confounding relationships of colony age and worker number on mean worker size or CV in worker size across the three resource environments.

**TABLE 2:** Size‐specific relationships of the smooth terms of colony age, the number of workers of each size (i.e., worker size composition, WSC), and standardized (“std”) WSC for each of the five vital rates relating to worker production

Response variable	Resource environment	Sample size	Smooth terms			Confounding effects^2^
			Colony age	WSC^1^	Std WSC^1^	
Egg production	Low	72	Concave	×, ×	×, ×	
	High‐low	74	Concave	±, ↑	±, ↑	Possibly
	High	65	Concave	±, ↑	±, ↑	No
Development time	Low	541	Multimodal	±, ↓	±, ↓	Possibly
	High‐low	974	Concave	±, ↓	±, ↓	Possibly
	High	1,108	Convex	±, ↓	±, ↓	Possibly
Larval survival	Low	3,521	Multimodal	±, ↕	±, ↕	Yes
	High‐low	6,045	Decreases	±, ↑	±, ↕	Yes
	High	5,364	Convex	−, ×	−, ↑	Yes
Mean callow size	Low	65	Decreases	±, ↑	±, ↑	Yes
	High‐low	59	Multimodal	±, ↑	±, ↑	Yes
	High	57	Multimodal	−, ×	−, ↑	Yes
CV in callow size	Low	65	Concave	×, ×	×, ×	
	High‐low	59	Multimodal	±, ↕	±, ↕	No
	High	57	Constant	×, ×	×, ×	

Note

Relationship descriptions provided are restricted over the observed range of worker body sizes and colony ages including days spent in the laboratory. Since colony age and population size are correlated, we were unable to determined which smooth term was driving these effects if both smooth terms have similar effects. Shaded grey cells had a significant fixed effect of colony ID on the parametric intercept in the GAM.

^1^ For WSC and std WSC, the first symbol refers to whether the relationship has a positive (+), negative (−), mixed (±), or no (×) per capita effect, and the second symbol refers to whether the relationship increases (↑), decreases (↓), both (↕), or has no effect (×) with worker size. Sample sizes are also provided for each of the five vital rates.

^2^ The column “confounding effects” describes whether both colony age and WSC had similar effects on the response variable when both smooth terms are significant.

## 3 RESULTS

Average worker size increased with available ambient resources (likelihood ratio (LR) test for models with and without treatment; *χ^2^* = 14,701, *df* = 3, *p* ≪ .001). Worker size was smallest in the low‐resource environment (mean and *SE* in IT span: 3.16 ± 0.049) and largest in the high‐resource environment (IT span: 3.68 ± 0.048) (multiple comparison of means between high and low; estimated difference, *E* = 0.52, *Z* = 7.5, *p* ≪ .001), with the high‐low‐resource environment being intermediate (IT span: 3.31 ± 0.049) (multiple comparison of means between high‐low and low: *E* = 0.14, *Z* = 2.1, *p* = .09; high and high‐low: *E* = 0.37, *Z* = 5.4, *p* ≪ s.001). These results broadly recapitulate results of previous analyses of the separate experiments as reported by Kerr et al. (2019) and Malfi et al. (2019) for the 2015 and 2016 data, respectively.

### 3.1 Daily egg production

**FIGURE 4:**
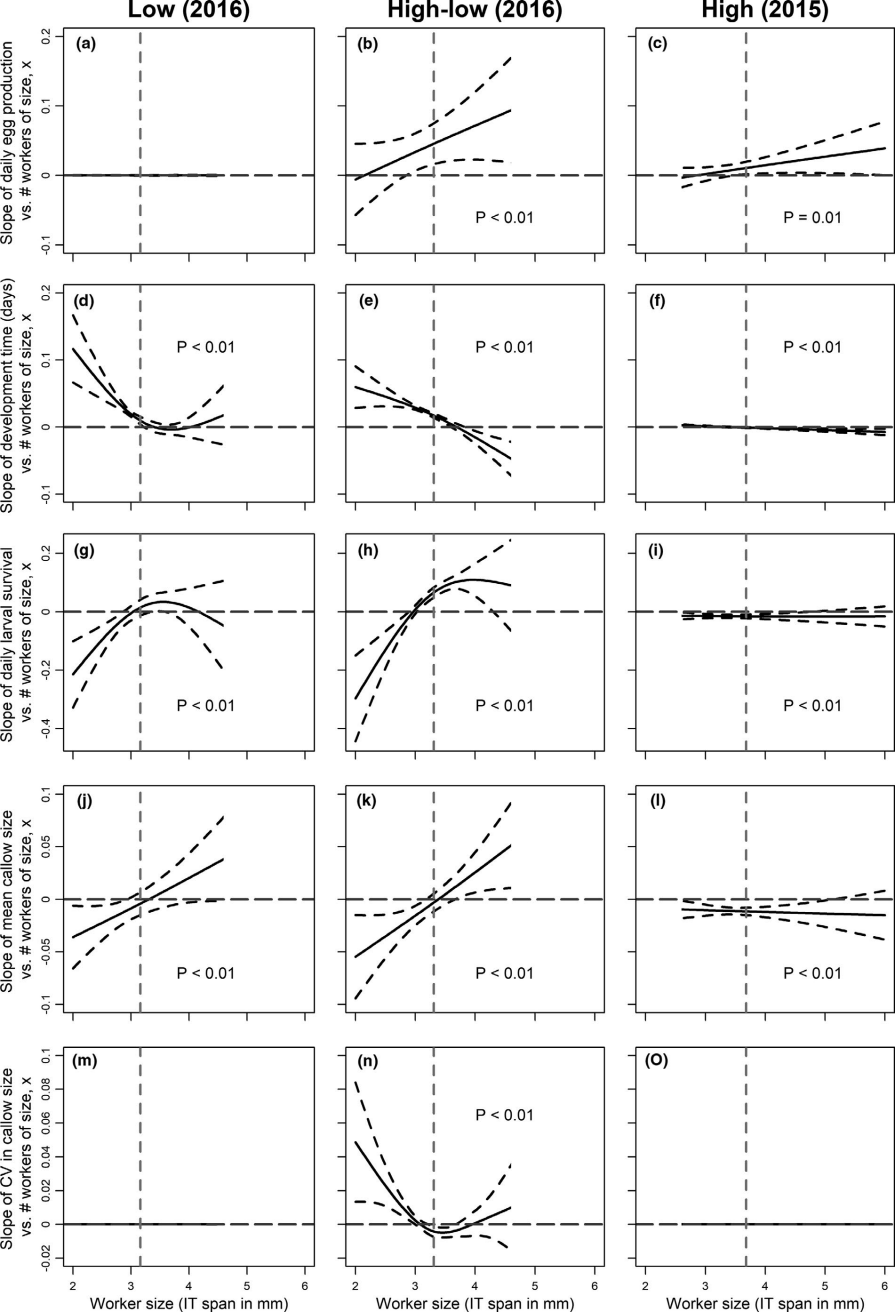
Generalized additive model results depicting the smooth function of the size‐specific slopes for all five vital rates relating to worker production versus the number of workers of size *x* for the low‐resource environment (left), high‐low‐resource environment (middle), and high‐resource environment (right). Dashed horizontal line at zero represent deviations from mean slope values, that is, slopes above the line means more workers of size *x* have positive impact on *Y*. Grey dashed vertical line represents the mean worker size for colonies in each of the resource environments. Plots with a significant smooth term of WSC are labeled with *p* < .01. Note different scales on the *Y*‐axes in each row

**FIGURE 5:**
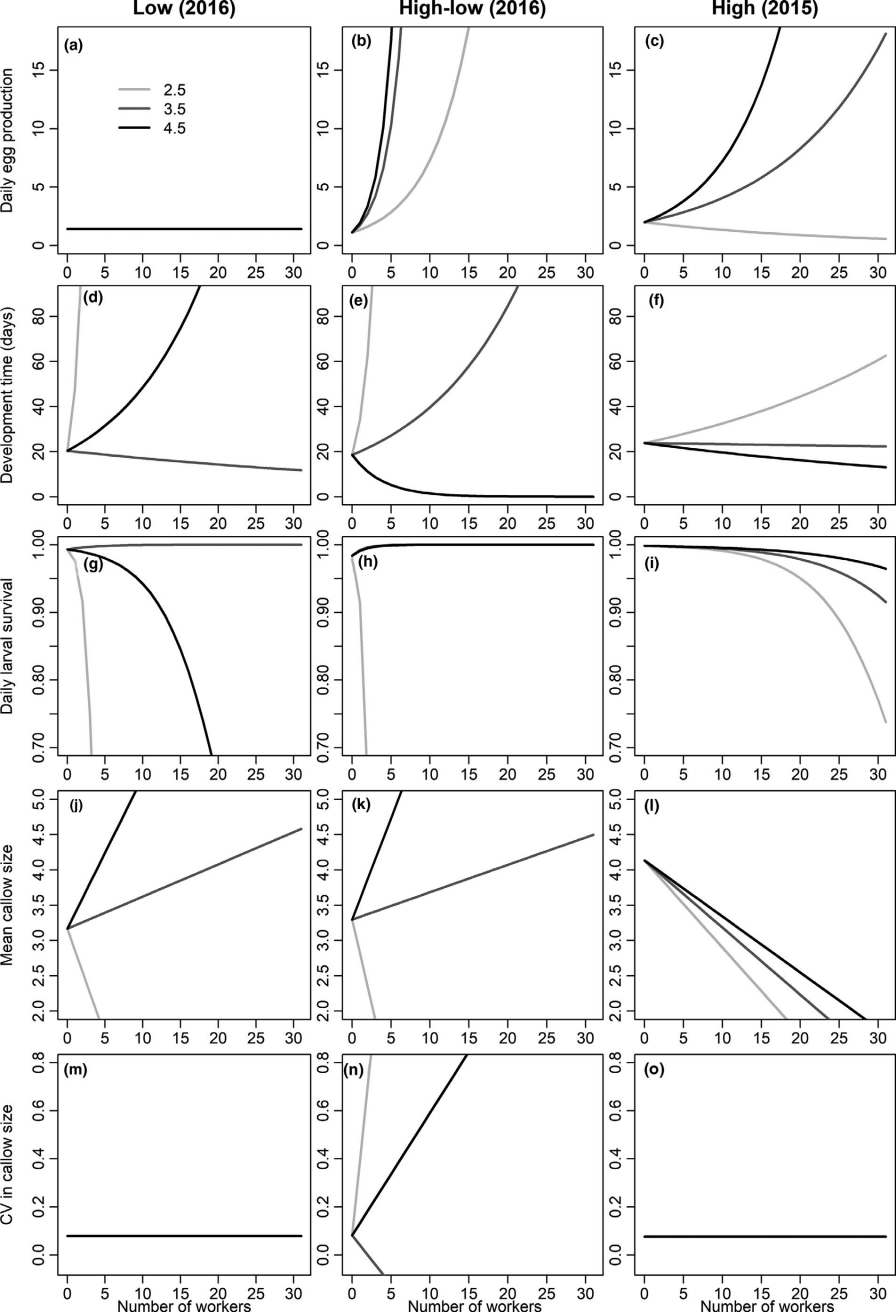
The relationship between number of workers of three observed worker sizes and the five vital rates relating to worker production across the three treatments. Three workers sizes range from the smallest size of 2.5 mm (light grey), intermediate size of 3.5 mm (dark grey), and largest size of 4.5 mm (black line) that are observed in colonies across all three treatments. Each of these lines represents the function defined by *x* = 2.5, 3.5, and 4.5 on the *x*‐axis of Figure 4. Parametric intercepts were used from the GAMs, and intercepts were averaged on the link function scale if the model had a significant fixed effect of colony

Worker size composition did not affect egg production in the low‐resource environment (Figure 4a; *χ* ^2^ = 6.3E−6, *e*.*df* = 4.2E−5, *p* = .75). More larger workers increased egg production in both the high‐low‐ and high‐resource environments (Figure 4b,c; *χ* ^2^ = 83.3, *e*.*df* = 2.8, *p* < .001, and *χ* ^2^ = 6.4, *e*.*df* = 1.3, *p* = .01 for high‐low and high (respectively)), but more larger workers had greater impact on egg production in the high‐low‐resource environment than in the constantly high‐resource environment (Table 1). To illustrate these differences for each vital rate, we plotted the lines predicted by FLMs for workers of different sizes (see egg production relationships in Figure 5a‐c).

### 3.2 Larval development time

Larval development time increased with more smaller workers in all three resource environments (Figure 4d‐f; LR test of smooth term versus constant: *χ* ^2^ = 124.6, *e.df* = 2.7, *p* < .001; *χ* ^2^ = 422.8, *e.df* = 2.4, *p* < .001; *χ* ^2^ = 21.4, *e.df* = 1.9, *p* < .001 for low, high‐low and high (respectively)). Worker size composition affected larval development time differently in each environment (Table 1). More larger workers decreased development time in both the high‐low‐ and high‐resource environments (Figure 4e,f) but not in the low‐resource environment (Figure 4d). However, these effects were negligible in the high‐resource environment compared to the low‐ and high‐low‐resource environments (Figure 5).

### 3.3 Larval survival

Larval survival decreased with more smaller workers in the low‐ and high‐low‐resource environments (Figure 4g,h; *χ* ^2^ = 18.9, *e.df* = 2.6, *p < *.001; *χ* ^2^ = 103.9, *e.df* = 2.6, *p < *.001 for low and high‐low (respectively)). The difference between the low and high‐low environments was not statistically significant (Table 1). Larval survival slightly decreased with more workers of all sizes in the high‐resource environment (Figure 4i; *χ* ^2^ = 29.1, *e.df* = 1.7, *p < *.001). This effect was negligible (Figure 5i), and this relationship for high‐resource colonies (i.e., colonies in 2015) differed significantly from both lower resource environments (i.e., treatments in 2016) (Table 1).

### 3.4 Callow size

In the low‐resource environment, mean callow size decreased with more smaller workers (Figures 4j and 5j; *F* = 3.3, *e.df* = 1.9, *p* = .007), but worker size composition was unrelated to CV in callow size (Figures 4m and 5m; *F* = 2.5E−6, *e.df* = 1.7E−5, *p* = .52). In the high‐low‐resource environment, mean callow size decreased with more smaller workers and increased with more larger workers (Figures 4k and 5k; *F* = 6.4, *e.df* = 2, *p* < .001), whereas more larger workers slightly decreased the CV in callow size (Figures 4n and 5n; high‐low ‐ *F* = 3.8, *e*.*df* = 3, *p* < .001). In the high‐resource environment, more workers of any size decreased the mean callow size (Figures 4l and 5l; *F* = 16.5, *e*.*df* = 1.7, *p* < .001), but worker size composition was unrelated to the CV in callow size (Figures 4o and 5o; high ‐ *F* = 5.2E−6, *e.df* = 4.6E−5, *p* = .59). The effect of worker size on mean callow size of new workers did not differ between the lower resource environments (i.e., 2016 treatments), but both differed from the high‐resource treatment (i.e., 2015 colonies) (Table 1). The effects of worker size on the CV in callow size differed among all three treatments (Table 1).

## 4 DISCUSSION

Size‐based contributions of bumblebee workers to worker production differed among vital rates and resource environments. Despite these differences, we never detected cases where smaller workers outperformed larger workers for vital rates relating to in‐nest tasks. Therefore, the fact that smaller workers remain in the nest is likely not due to their superior skill at those in‐colony tasks (Jandt & Dornhaus, 2014). Instead, colonies with more larger workers often had greater worker production compared to colonies with smaller workers. This pattern is similar to many performance‐based (Goulson et al., 2002; Ings, 2007; Kapustjanskij et al., 2007; Peat & Goulson, 2005; Spaethe et al., 2007; Spaethe & Weidenmüller, 2002) and manipulative experiments (Cnaani & Hefetz, 1994). However, we found the opposite effect in two cases: more workers of any size slightly decreased both larval survival and mean callow size in the high‐resource environment. We discuss each result in turn below, as well as some advantages and limitations of functional linear models.

For two vital rates, larval survival and mean callow size, both treatments applied in 2016 differed from 2015, and not from each other. Therefore, these differences could be due to other features that differed among the sites where the two experiments were conducted or conditions in the 2 years. For example, the site of the 2016 experiment was an agricultural field in an agricultural landscape. The field of the experiment was used only for growing flowers to create the “high” resource pulse in the “high‐low” treatment. Nevertheless, pesticides and other factors (such as nest temperatures) may have differed between the two landscape contexts. In general, conditions for bumblebees in the 2016 experiment appeared to be more stressful than conditions in the 2015 experiment. Although the results are not uniquely attributable to floral resources, our analyses provide a reasonable test of size‐based differences under relatively low‐ to high‐stress levels.

### 4.1 Functional implications of worker size distribution

Across social organisms, the number of offspring produced often increases with the number of helpers (Biedermann & Tab orsky, 2011; Brown et al., 1982; Malcolm & Marten, 1982; Young et al., 2015), particularly when resources are high (Doolan & Macdonald, 1997; Harrington et al., 1983). We found a similar per capita effect on colony egg production in both our high‐low‐ and high‐resource treatments, yet FLMs also revealed that in these environments more larger workers increased colony egg production relative to more smaller workers. Laboratory studies of bumblebees have shown that colonies consisting of only larger workers produce more eggs than colonies consisting of only smaller workers (Cnaani & Hefetz, 1994). Larger workers are known to return more resources to the colony (Goulson et al., 2002; Kerr et al., 2019), but they are less resilient against starvation (Couvillon & Dornhaus, 2010). This trade‐off might explain why larger workers increased colony egg production only in the high‐low‐ and high‐resource environment but not in the low‐resource environment. The opposite effect has been found in a fire ant, *Solenopsis invicta*, where monomorphic colonies of large workers produced almost no brood compared to monomorphic colonies of small workers (Porter & Tschinkel, 1985). However, the size‐based roles of workers in these two eusocial insects differ. Larger bumblebees are foragers (Cumber, 1949; Goulson, 2009; Goulson et al., 2002), but smaller fire ant workers do most of the foraging and feeding (Cassill & Tschinkel, 1999; Wilson, 1978). Larger fire ant workers live longer than smaller workers (Calabi & Porter, 1989; Porter & Tschinkel, 1985), which is the opposite of bumblebee workers (Kerr et al., 2019; da Silva‐Matos & Garofalo, 2000). Therefore, the general mechanism may be similar, despite contrasting patterns.

The smallest observed workers had negative effects on both development time and larval survival in the low‐ and high‐low‐resource environments; note that this worker size was not present in the high‐resource colonies. In bumblebees, there seems to be a resource‐driven trade‐off between provisioning for developing larvae and production of new eggs when resources are low. For example, in the low‐resource environment, egg‐laying did not depend on the number of large workers. In contrast, in higher resource environments, the number of eggs laid increased with more larger workers. This contrast suggests that workers in the low‐resource environment are allocating more resources to maintaining larval survival and development time, rather than supporting more workers. Results for small workers in the lower resource environments are similar to those for cooperative breeding species, in which the presence of more helpers often reduces offspring survival when resources are low (Harrington et al., 1983; Woodroffe & Macdonald, 2000). These negative impacts of helpers in cooperative breeding species may be due to them shifting efforts toward increasing their own survival (Bruintjes et al., 2010), which seems less likely in bumblebees because workers are nonreproductive. Indeed, bumblebee workers are reported to switch from nursing to foraging tasks when resources are low (Cartar, 1992), indicating that workers overall increase (not decrease) cooperative efforts. Additionally, bumblebee workers predominantly feed on nectar and larvae predominantly feed on pollen (Goulson, 2009; Plowright & Pendrel, 1977), which may reduce competition among siblings and enhance cooperative behaviors. It would be interesting to monitor foraging behavior of bumblebee workers during resource dearths, that is, changes in nectar versus pollen collection rates, to better understand their cooperative efforts.

Across our three environments, observed average size of all workers decreased in colonies with less available resources. In the high‐resource environment, more workers of any size decreased the size of callow workers. Worker size is known to decrease with colony age (Couvillon et al., 2010), which correlated with colony size. In the low and high‐low‐resource environments, more smaller workers resulted in callow workers of smaller sizes and more larger workers resulted in callow workers of larger sizes. Bumblebee workers have been recorded to be smaller on average in simple, intensively managed landscapes (Persson & Smith, 2011). Laboratory experiments also show that colonies produce smaller workers during food shortages (Schmid‐Hempel & Schmid‐Hempel, 1998). The correlation between worker size distribution and callow worker size in the low‐ and high‐low‐resource environment suggests that stressful resource conditions may produce a negative feedback loop, where colonies of smaller workers cannot properly feed and care for brood (Cartar & Dill, 1991) causing the emergence of smaller callow workers. Therefore, the cost and benefits of helpers within social groups may often regulate the traits of individuals (e.g., sex ratios, worker sizes) that are expressed (Griffin et al., 2005). Functional linear models are only a correlative technique, so an alternative shared driver could be shifting the size distribution toward smaller workers. For example, lower resources could cause differential mortality of larger workers due to starvation (Couvillon & Dornhaus, 2010) and cause larvae to develop into smaller callow workers because of fewer resources brought back by the remaining workers. Laboratory monomorphic colonies consisting of only small or large workers had no difference in the mean and variance in callow size when supplied with abundant resources (Cnaani & Hefetz, 1994). If these laboratory colonies had to forage for resources and still produced workers of similar sizes, then we might be able to determine whether a shared driver is most likely causing these effects in our study.

### 4.2 Functional linear models as a statistical approach in ecology

Previously, FLMs have been used to evaluate the lagged effects of environmental drivers on plant population dynamics (Teller et al., 2016; Tenhumberg et al., 2018). Here, we extend the use of FLMs to evaluate the size‐based contribution of workers in bumblebee colonies. FLMs could be applied to understanding many high‐dimensional social systems. For example, they could be used to explore the contributions of trait‐based sociality, such as the contributions of age polyethism within social groups of different taxa and levels of sociality, including eusocial honey bees (Seeley & Kolmes, 1991), semisocial mole rates (Jarvis, 1981; Zöttl et al., 2016), and cooperative breeding meerkats (Clutton‐Brock, Brotherton, et al., 2001) or cichlid fish (Bruintjes & Tab orsky, 2011). In the African mole rat, larger groups had higher rates of offspring recruitment (Young et al., 2015) and cooperative behaviors were found to increase with age (Zöttl et al., 2016). Therefore, FLMs might be able to determine how vital rates (e.g., offspring recruitment) differ with the number of helpers of different ages for the African mole rate. FLMs provide an alternative way to study these high‐dimensional ecological systems using field observational data, particularly where manipulative experiments may not be possible.

Correlative techniques, such FLMs, provide a valuable complement to many manipulative experiments that aim to test similar hypotheses. However, these separate approaches have their own set of advantages and limitations that need to be considered when making conclusions from these models. For example, FLMs can be data‐heavy (e.g., 20–25 independent observations of the signal and response; Teller et al., 2016); only inform us about correlations and not causations; and may have collinear predictors that obscure the true driver of these responses. Collinearity is not specific to FLMs but is equally problematic for many simple (e.g., multiple regression) and complex statistical techniques (e.g., structural equation models). To date, only two studies have reported applying functional smoothing approaches to high‐dimensional ecological systems by exploring how lagged environmental drivers influence plant performance (Teller et al., 2016; Tenhumberg et al., 2018). Teller et al. (2016) predicted how lagged effects of past precipitation and local competition influenced plant growth and survival; however, they would not be able to parse out the true driver of plant performance if density and precipitation covaried across some gradient. When exploring the trends and collinearity for these several vital rates (Appendix S4), two of four vital rates (Table 2) had confounding effects of colony age and size composition suggesting that either or both might be driving these trends (Table 2). When using simple or complex correlative methods, it is important to explicitly evaluate the collinearity of predictor variables as we have demonstrated here.

### 4.3 Summary

Overall, we found that the advantages and disadvantages of workers of different sizes on worker production only became apparent when exploring these effects across these three different resource environments. We also found that bumblebee colonies shifted their worker size distribution across these resource environments. Among eusocial insects, caste size polymorphism is hypothesized to be an adaption to expand accessibility of resources, such as seed size in ants (Davidson, 1978; Retana & Cerdá, 1994; Traniello & Beshers, 1991) and flower size in bumblebees (Peat et al., 2005). However, the shift in worker size distribution across these resource environments could have emerged from the lower tolerance of larger workers to starvation (Couvillon & Dornhaus, 2010). Prior to this study, quantifying the contribution of individuals in social groups has been challenging. Here, we demonstrate that functional linear models have the potential to evaluate observational data for complex, trait‐based life histories of social organisms. As such, they provide a valuable complement to the constraints of experimental work and a mechanism to focus hypotheses for further experimental studies.

## CONFLICT OF INTEREST

We have no conflicts of interest to declare.

## AUTHOR CONTRIBUTIONS

**Natalie Z. Kerr:** Conceptualization (equal); data curation (lead); formal analysis (equal); investigation (equal); methodology (equal); project administration (equal); visualization (lead); writing‐original draft (lead); writing‐review & editing (equal). **Rosemary L. Malfi:** Data curation (supporting); investigation (supporting); project administration (equal); resources (equal); writing‐review & editing (equal). **Neal M. Williams:** Funding acquisition (lead); investigation (supporting); project administration (equal); resources (equal); supervision (lead); writing‐review & editing (equal). **Elizabeth E. Crone:** Conceptualization (equal); formal analysis (equal); funding acquisition (lead); investigation (equal); methodology (equal); project administration (equal); supervision (lead); visualization (supporting); writing‐original draft (supporting); writing‐review & editing (equal).

## Supporting information

Appendix S1

Supplementary Material

## Data Availability

Demographic data: Dryad https://doi.org/10.5061/dryad.mkkwh70zk.
