# Supplementary material for: Larger workers outperform smaller workers across resource environments: An evaluation of demographic data using functional linear models
Source: Ecol Evol. 2021 Feb 11;11(6):2814–27. doi: 10.1002/ece3.7239 (PMC7981203; doi:10.1002/ece3.7239)
Supplement: Supplementary file 2 — Supplementary Material [file ECE3-11-2814-s001.docx]

SUPPORTING INFORMATION

**Figure S1.** Google earth aerial images of the 2015 and 2016 field sites at UC Davis.

**Figure S2.** Scatterplot matrix of mean worker size, CV in worker size, colony age, and colony size for colonies in the low resource environment.

**Figure S3.** Scatterplot matrix of mean worker size, CV in worker size, colony age, and colony size for colonies in the high-low resource environment.

**Figure S4.** Scatterplot matrix of mean worker size, CV in worker size, colony age, and colony size for colonies in the high resource environment.

**Appendix S1.** RMarkdown file containing code for running functional linear models.

**Appendix S2.** Exploring the effects of worker size composition on vital rates when standardized by worker production costs.

**Appendix S3.** Exploring effects of colony age on vital rates using GAMs.

**Appendix S4.** Evaluating confounding effects of colony age and worker size composition on vital rates affecting worker production.

**Figure S1.** Google earth aerial images of the field sites at UC Davis during the experiment periods. A larger landscape view (left) and smaller scale view (right) of each of the study sites. The 2015 colonies (a-b) were located at Harry H. Laidlaw Honey Bee Research (red box) at UC Davis, where the surrounding landscape was agricultural land, floral research plots (orange boxes), and 0.2 ha bee-friendly garden (blue box). The 2016 colonies were located (c-d) in agricultural fields on UC Davis Experimental Farm property. The yellow box in Fig 2d identifies supplemental forb plantings used to add resources to high-low colonies in this experiment.


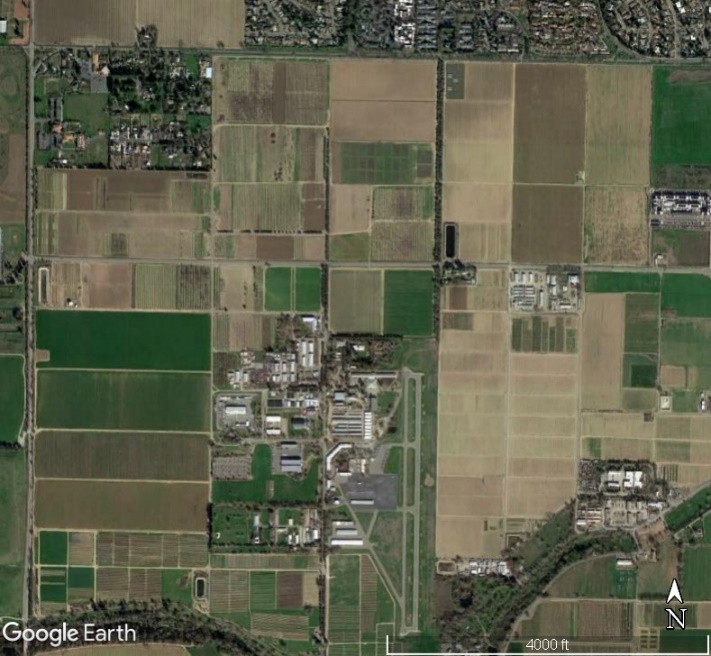

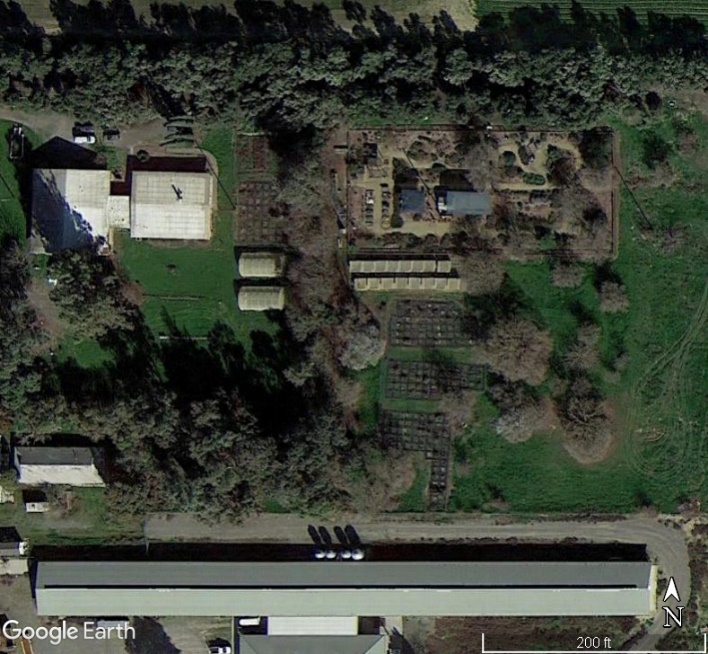


(a)

(d)

(c)

(b)


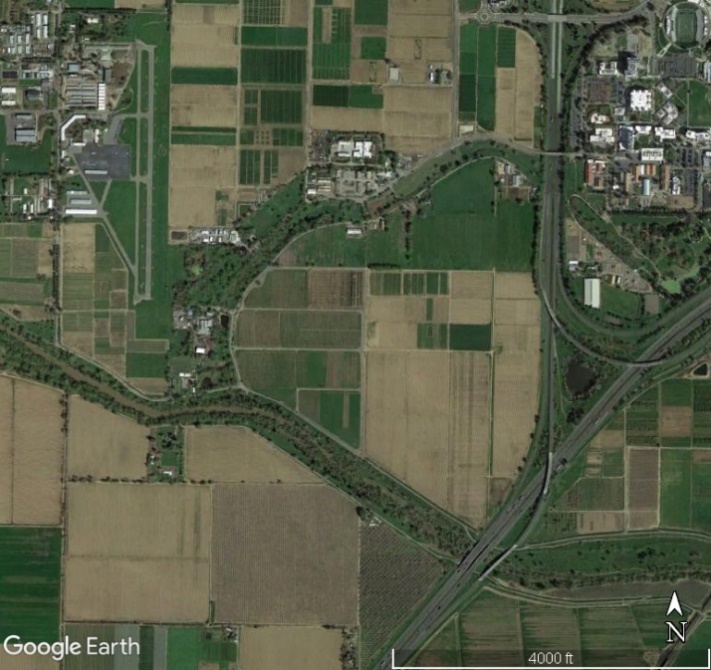

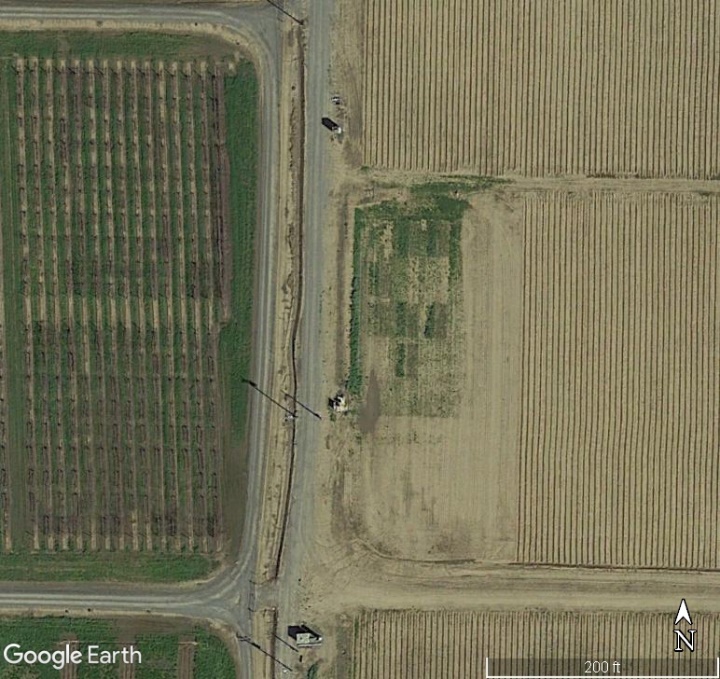


**Figure S2.** Scatterplot matrix of mean and coefficient of variation (CV) in worker size measured as intertegular span in mm (ITS), colony age, and colony size of colonies in the low resource environment. The leading diagonal contains a histogram a of each variable, the upper diagonal panels contains the correlation coefficient between each pairwise comparison, and the lower diagonal panel contains a scatterplot with the mean and 95% confidence intervals between each pair.

**
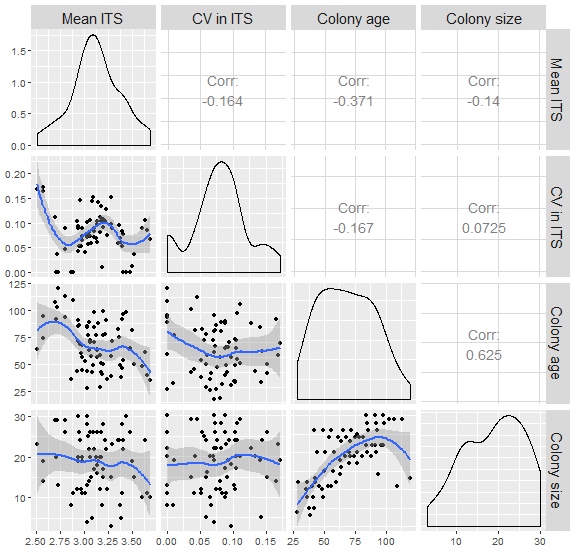
**

**Figure S3.** Scatterplot matrix of mean and coefficient of variation (CV) in worker size measured as intertegular span in mm (ITS), colony age, and colony size of colonies in the high-low resource environment. The leading diagonal contains a histogram a of each variable, the upper diagonal panels contains the correlation coefficient between each pairwise comparison, and the lower diagonal panel contains a scatterplot with the mean and 95% confidence intervals between each pair.


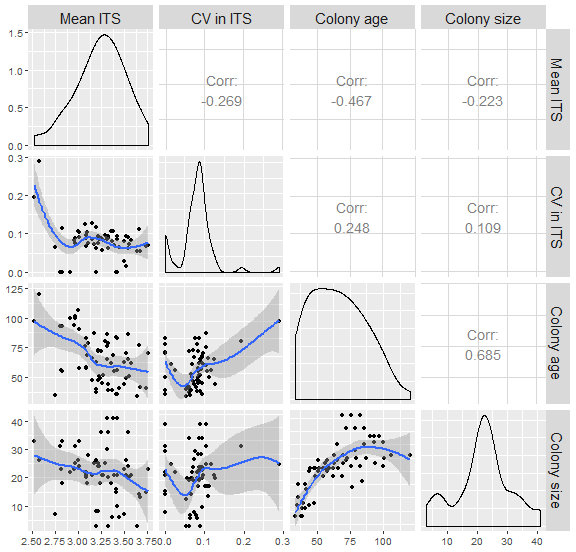


**Figure S4.** Scatterplot matrix of mean and coefficient of variation (CV) in worker size measured as intertegular span in mm (ITS), colony age, and colony size of colonies in the high resource environment. The leading diagonal contains a histogram a of each variable, the upper diagonal panels contains the correlation coefficient between each pairwise comparison, and the lower diagonal panel contains a scatterplot with the mean and 95% confidence intervals between each pair.


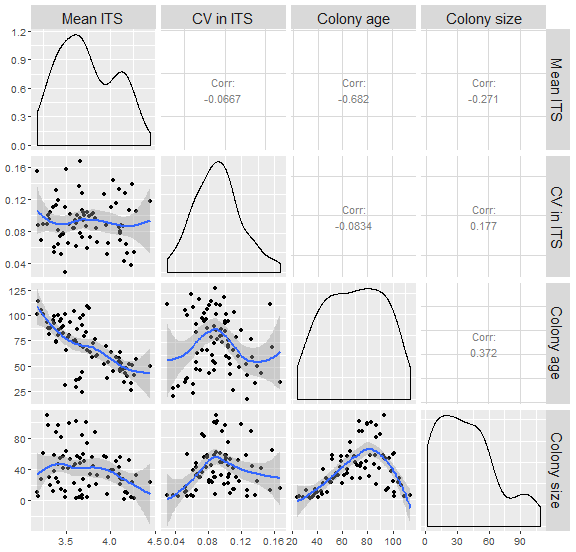


**Appendix S2.** Exploring the effects of worker size composition on vital rates when standardized by worker production costs.

Methods

Even if larger workers contribute more to the production of new workers on a per capita basis, smaller workers may have more benefits after accounting for differences in their production costs (c.f. Kerr, Crone & Williams 2019). Therefore, in addition to presenting FLMs as fitted (see Fig. 3), we also adjusted our worker size composition functions to reflect a constant mass of workers of each size (see Fig. 4). For example, if we found that more larger workers increased egg production, colonies producing only larger workers would pay a cost of having fewer workers due to their higher respective production costs. To adjust these functions by size-based production costs of workers, we converted slopes (change in response per worker) to change in response per mass. Specifically, we divided size-specific slopes,  *(s_x_)*, by mass per worker in each size class, using the mass function reported by Kerr et al. (2019). Here, we present FLM results that account for worker production costs, to evaluate whether the size-based cost of worker biomass would affect contributions of different-sized workers.

Results

*Egg production*

When accounting for production costs of larger workers, more larger workers still increased egg production in both high-low and high resource environments. For workers > 4 mm, the effect of worker size composition on egg production was negligible (Fig. S2.1b-c).

*Larval development time*

Larval development time increased even more with more smaller workers in the low and high-low resource environments when accounting for production costs (Fig. S2.1d-e). Larvae took slightly longer to develop with more larger workers compared to more intermediate-sized workers in the low resource environment (Fig. S2.1d), but larval development time still decreased with more larger workers in the high-low environment (Fig. S2.1e). More small workers increased development time in the high resource environment when accounting for production costs (Fig. S2.1f), but the magnitude of this effect was negligible for observed worker sizes (see Fig. S5f).

*Larval survival*

Larval survival decreased with more smaller workers in the low and high-low resource environments, even after accounting for production costs (Fig. S2.1g-h). More smaller workers decreased larval survival more in the high-low environment compared to the low resource environment. More workers of any size decreased larval survival in the high resource environment (Fig. 4i), but smaller workers decreased larval survival more than larger workers when accounting for production costs (Fig. S2.1i). However, the magnitude of this effect was negligible over the realized range of worker size distributions (Fig. 5i).

*Callow size*

More smaller workers decreased the mean callow size in the low resource environment (Fig. 4j); an effect that was exaggerated when we accounted for production cost (Fig. S2.1j). Worker size composition had no effect on the CV in callow size in the low resource environment, even when accounting for production costs (Fig. S2.1m).

More smaller workers also decreased the mean callow size in the high-low resource environment (Fig. 4k); an effect that was also exaggerated when accounting for production costs, even more so than the low resource environment (Fig. S2.1j-k). More smaller workers increased the CV in callow size in high-low resource environment with intermediate-sized workers decreasing and large workers slightly increasing the CV in callow size (Fig. 4n); an effect that was exaggerated when accounting for production costs (Fig. S2.1n).

More workers of any size still decreased the mean callow size in the high resource environment (Fig. S2.1), but more smaller workers decreased mean callow size more than larger workers when accounting for production costs (Fig. 2.1l). No effects of worker size composition on CV in callow size were seen in the high resource environment (Fig. 2o), but more workers of any size slightly decreased the CV in callow size when accounting for production costs (Fig. S2.1o).

**
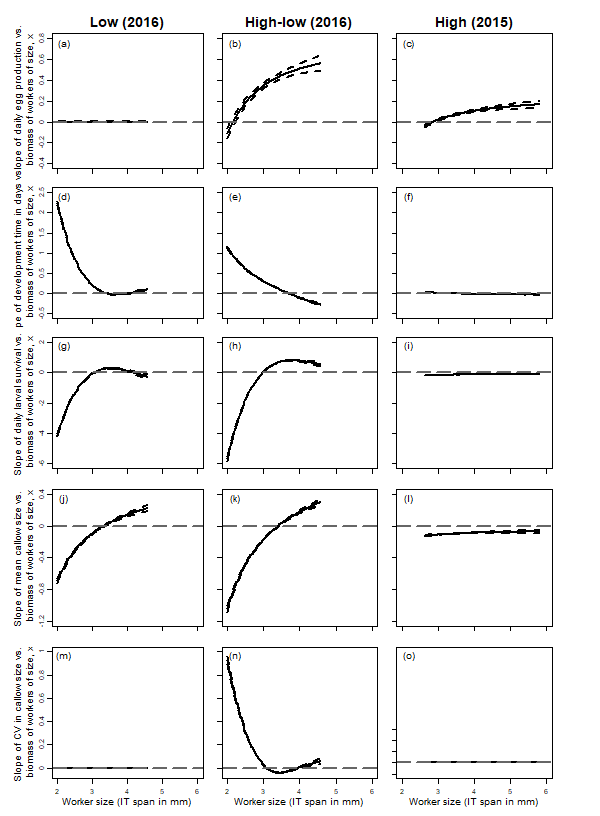
**

**Figure S2.1.** Generalized additive model results standardized by worker production costs (i.e. worker biomass) depicting the smooth function of the slopes for all five vital rates versus the number of workers of size *x* per worker biomass as a function of worker size *x* for the low (left), high-low (middle), and high (right) resource environments. Dashed horizontal line at zero represent deviations from mean slope values per worker biomass, i.e. slopes above the line means more workers of size x have positive impact on Y. Note different scales on the Y-axes in each row.

**Appendix S3.** Exploring effects of colony age on vital rates using GAMs.

Methods

In addition to the FLMs for worker size composition (see main text), we fit a second set of GAMs predicting each response as a smooth covariate of colony age. For new eggs laid and larval development time, we knew when eggs first appeared and used the colony age at this time of first detection. For larval survival, we used colony age for each week of the census that they were alive until they either died or eclosed. We estimated time of first detection for each callow worker in each colony, and we used the date of emergence minus average development time for each colony to estimate colony age. We used the same probability distributions as the worker size composition FLMs for evaluating colony age effects. We did not restrict the number of knots for colony age GAMs since lab and field conditions may alter the effect of colony age on vital rates.

Results

Here, we only present results when colonies were outside in the field, in their respective resource environments, rather than when colonies were inside the laboratory prior to being exposed to these different resource environments.

In the low and high-low resource environments, daily egg production remained mostly constant with increasing colony age until 60 days old at which point egg production steadily declined (Fig. S3.1a-b; significance of colony age smooth term: low - *χ^2^* = 34.2, *e.d.f*. = 1.9, *P* < 0.001; high-low - *χ^2^* = 83.3, *e.d.f.* = 2.8, *P* < 0.001). For the high-low resource environment, this decline happened approximately when the early season pulse ended (Fig. 3.1b). In the high resource environment, egg production slightly increased with colony age until 60 days, then slightly decreased with increasing colony age (Fig. S3.1c; *χ^2^* = 11.7, *e.d.f.* = 1.8, *P* = 0.002).

In the low resource environment, larval development time remained somewhat constant as the colony aged (Fig. S3.1d; *χ^2^* = 199.2, *e.d.f.* = 3.9, *P* < 0.001). In the high-low environment, larval development increased with colony age until approximately 60 days and then decreased (Fig. S3.1e; *χ^2^* = 571, *e.d.f.* = 3, *P* < 0.001); this transition is likely to align with the end of the early season pulse since colony age was defined for development time when cells first appeared as eggs rather than when they were feeding off pollen as larvae. Larval development time remained constant with colony age in the high resource environment (Fig. S3.1f; *χ^2^* = 8.8, *e.d.f.* = 1.5, *P* = 0.004).

In the low resource environment, larval survival slightly increased until approximately 60 days, then decreased (Fig. S3.1g; *χ^2^ =* 187, *e.d.f.* = 3.7, *P* < 0.001). In the high-low resource environment, larval survival decreased with increasing colony age (*χ^2^ =* 526.2, *e.d.f.* = 1.9, *P <* 0.001), particularly after the early season pulse (Fig. S3.1h). In the high resource environment, larval survival decreased until 80 days then slightly increased (Fig S3.1i; *χ^2^ =* 57.3, *e.d.f.* = 2.6, *P <* 0.001).

Mean callow size decreased with colony age in both low and high-low resource environments (Fig. S3.1j-k; low - *F* = 4.3, *e.d.f.* = 1.1, *P* = 0.003; high-low - *F* = 8.5, *e.d.f.* = 2.5, *P* < 0.001), particularly for the high-low resource environment when the early season pulse ended (Fig. S3.1k). Mean callow size remained approximately constant after colonies were placed in the high resource environment (Fig. S3.1l; *F* = 26.4, *e.d.f.* = 3.2, *P* < 0.001). For the low and high resource environment, CV in callow size remained mostly constant with colony age (Fig. S3.1m,o; low - *F* = 4.8, *e.d.f.* = 1.8, *P* = 0.006; high - *F* = 8.2E-5, *e.d.f.* = 2.6E-4, *P* = 0.44), whereas CV in callow size increased in the high-low resource environment after the early season pulse (Fig. S3.1n; *F* = 5.7, *e.d.f.* = 3.4, *P* < 0.001).

**
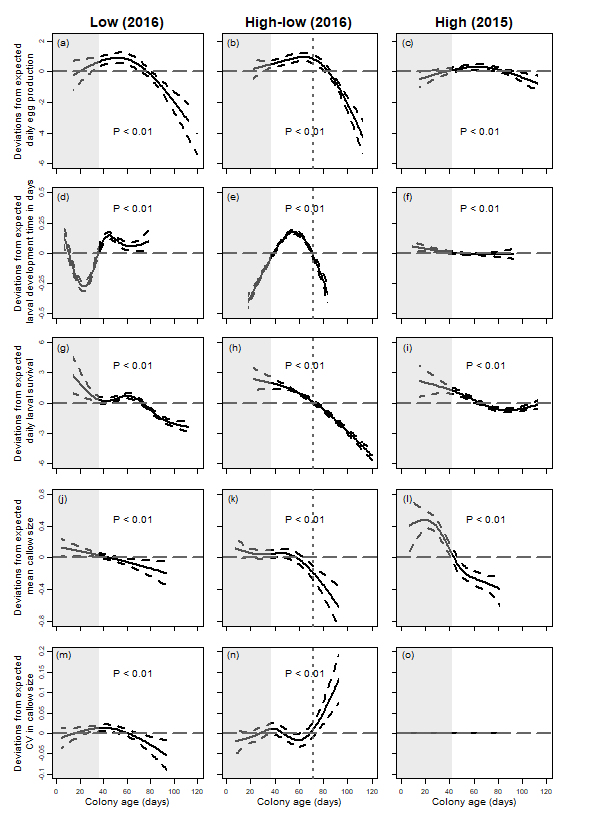
Figure S3.1.** Generalized additive model results depicting the smooth function of the deviations from expected values for all five vital rates as a function of colony age in days for the low (left), high-low (middle), and high (right) resource environments. Dashed horizontal line represent deviations from mean values, i.e. values above the line have positive impact on Y and values below the line have negative effects. Dotted vertical line represents the mean colony age at when the early season pulse ended (and transitioned to ambient resources) for the high-low resource environment. Shaded and unshaded areas in the plot represent when the colonies were in the laboratory and outside in the field, respectively. Because all colonies experienced similar conditions in the lab, we focus on results during the time when they were in field environments (non-shaded area). Plots with a significant smooth term of colony age are labeled with P < 0.01. Note different scales on the Y-axes in each row.

**Appendix S4.** Evaluating confounding effects of colony age and worker size composition smooth terms on five vital rates affecting worker production.

1. *Egg production*

Low: Colony age had no significant effects on egg production in the low resource environment. Therefore, we did not need to evaluate confounding effects for this vital rate.

High-low: Colony age and worker size composition both had significant effects on egg production in the high-low resource environment. When exploring confounding effects, we found that egg prodution is the highest when the colony was 40-80 days old (Fig S4.1c), which slightly overlaps when colony size was the highest after 70 days old (Fig S4.1a). However, more larger workers contribute the most towards egg production (Fig S4.1d), which were found at younger colony ages (Fig S4.1b). Regardless, more workers of most worker sizes increases egg production (Fig S4.1d) suggesting that these smooth terms might be confounded.

**
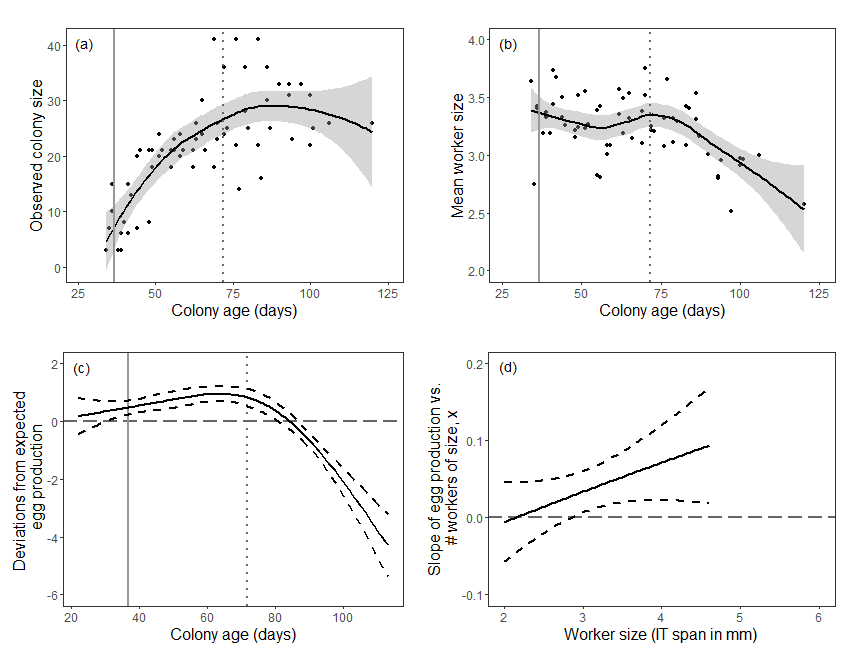
**

**Figure S4.1.** The (a) observed colony size (i.e. number of workers) and (b) mean worker size plotted against colony age in the high-low resource environment. Smooth components of generalized linear models evaluating the (c) deviations from expected egg production as a function of colony age and (d) the slope of egg production vs. # of workers as a function of worker size *x* for the high-low resource environment. The solid grey line represents the mean age when colonies were relocated from the laboratory into the field, and the dotted vertical line represents the end of the early season pulse for the high-low resource environment.

High: Colony age and worker size composition both had significant effects on egg production in the high-low resource environment. More larger workers contribute the most towards egg production (Fig S4.2d), which were found at younger colony ages (Fig S4.2b). We also found that egg prodution is the highest at colony age 60 (Fig S4.2c), but colony size was the highest at 80 days old (Fig S4.2a). Therefore, colony age and WSC do not have confounding effects.

*
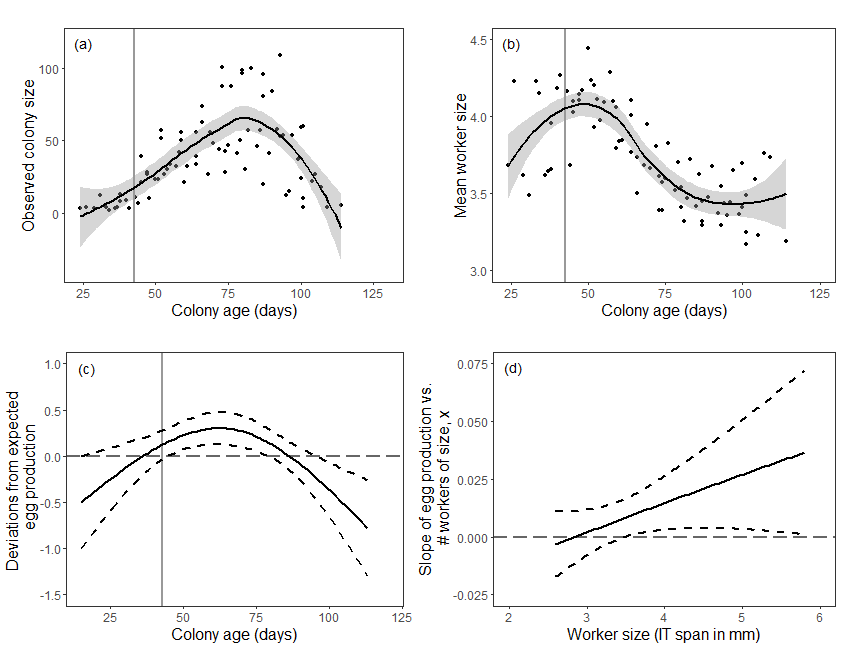
*

**Figure S4.2.** The (a) observed colony size (i.e. number of workers) and (b) mean worker size plotted against colony age in the high resource environment. Smooth components of generalized linear models evaluating the (c) deviations from expected egg production as a function of colony age and (d) the slope of egg production vs. # of workers as a function of worker size *x* for the high resource environment. The solid grey line represents the mean age when colonies were relocated from the laboratory into the field.

1. *Development time*

Low: Colony age and worker size composition both had significant effects on larval development time in the low resource environment. More smaller workers increased development time (Fig S4.3d), and worker size seems to be slightly lower at older colony ages (Fig S4.3b) when colony size and development time were high (Fig S4.3a,c) but not the highest. Therefore, colony age and WSC might have confounding effects. However, these colony age patterns on development time are likely attributed to lab conditions. Due to higher resource availability in the lab compared to the field, more larger workers eclosed and larvae developed faster. Since development time stabilized once in the field, we would contribute these early colony age patterns to worker size and feeding regiments in the lab.


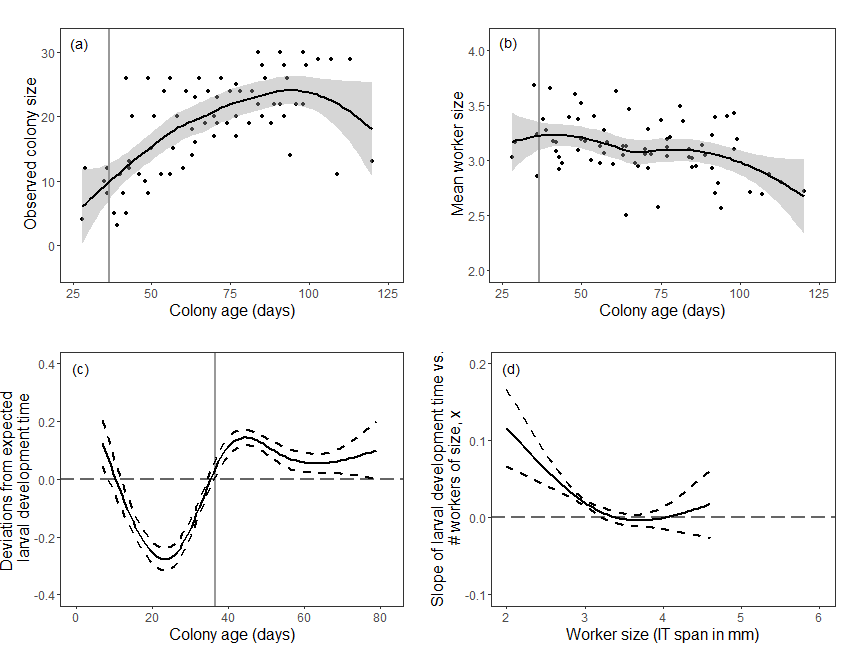


**Figure S4.3.** The (a) observed colony size (i.e. number of workers) and (b) mean worker size plotted against colony age in the low resource environment. Smooth components of generalized linear models evaluating the (c) deviations from expected egg production as a function of colony age and (d) the slope of egg production vs. # of workers as a function of worker size *x* for the low resource environment. The solid grey line represents the mean age when colonies were relocated from the laboratory into the field.

High-low: Colony age and worker size composition both had significant effects on larval development time in the high-low resource environment. Development time was the highest around 60 days old (Fig S4.4c), which is the intermediate colony age. More smaller workers increased development time (Fig S4.4d), and worker size was the lowest at older colony ages (Fig S4.4b) when development time was the low (Fig. S4.4c). More larger workers decreased development time (Fig. S4.4c), and worker size was the highest at younger colony ages (Fig. S4.4b) when development time was low (Fig. S4.4c). Therefore, colony age and WSC might have confounding effects. However, similar to the low resource environment, the feeding conditions in the laboratory might have decreased development and increased worker size.


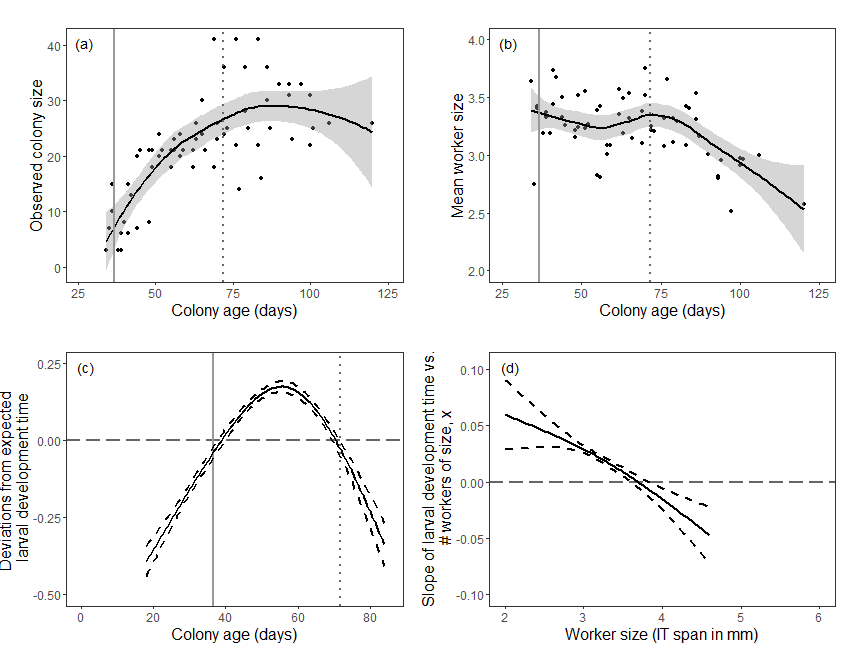


**Figure S4.4.** The (a) observed colony size (i.e. number of workers) and (b) mean worker size plotted against colony age in the high-low resource environment. Smooth components of generalized linear models evaluating the (c) deviations from expected larval development time as a function of colony age and (d) the slope of larval development time vs. # of workers as a function of worker size *x* for the high-low resource environment. The solid grey line represents the mean age when colonies were relocated from the laboratory into the field, and the dotted vertical line represents the end of the early season pulse for the high-low resource environment.

High: Colony age and worker size composition both had significant effects on larval development time in the high resource environment. More smaller workers increased development time (Fig. S4.5d), and worker size was lowest at older colony ages (Fig. S4.5b) when colony size was lower and development time was lower (Fig. S4.5a,c). More larger workers decreased development time (Fig. S4.5d), and worker size was the highest around colony age of 40-60 days (Fig. S4.5b) when colony size and development time was low (Fig. S4.5a,c). Therefore, colony age and WSC might have confounding effects.


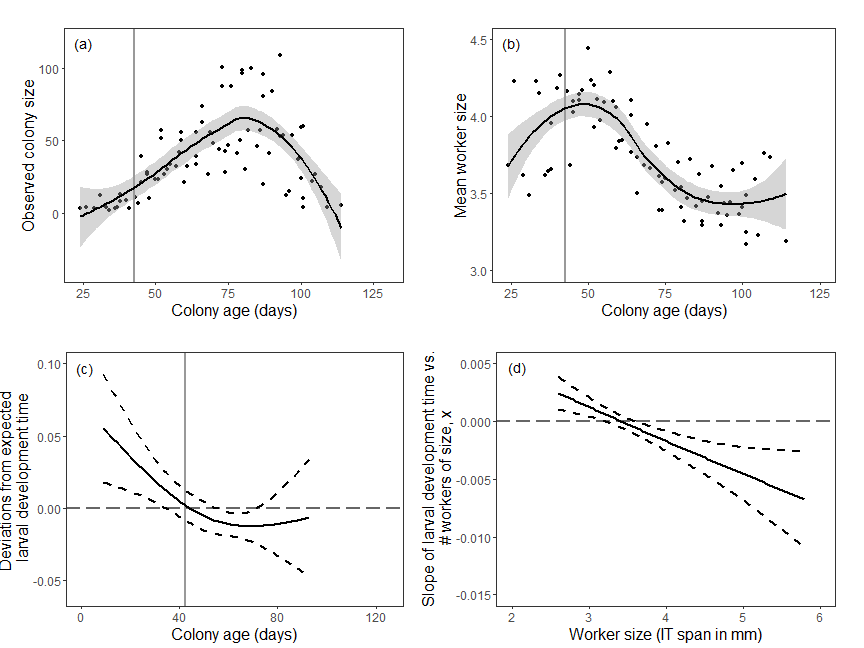


**Figure S4.5.** The (a) observed colony size (i.e. number of workers) and (b) mean worker size plotted against colony age in the high resource environment. Smooth components of generalized linear models evaluating the (c) deviations from expected larval development time as a function of colony age and (d) the slope of larval development time vs. # of workers as a function of worker size *x* for the high resource environment. The solid grey line represents the mean age when colonies were relocated from the laboratory into the field.

1. *Larval survival*

Low: Colony age and worker size composition both had significant effects on larval survival in the low resource environment. Larval survival decreased with more smaller workers (Fig S4.6d), and worker size seemed to be the lowest at older colony ages (Fig S4.6b) when colony size was highest and larval survival was the lowest (Fig. S4.6a,c). Therefore, colony age and WSC have confounding effects.


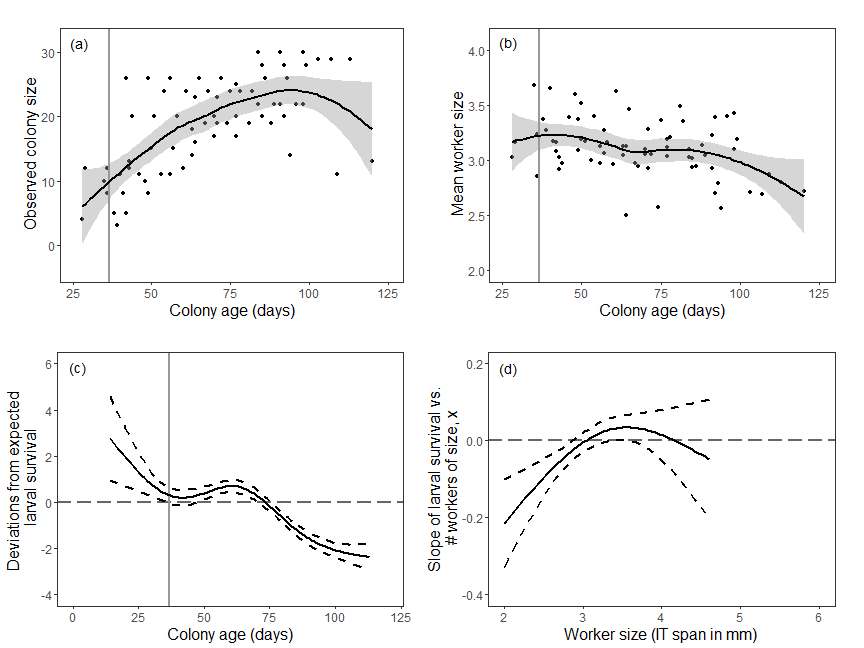


**Figure S4.6.** The (a) observed colony size (i.e. number of workers) and (b) mean worker size plotted against colony age in the low resource environment. Smooth components of generalized linear models evaluating the (c) deviations from expected larval survival as a function of colony age and (d) the slope of larval survival vs. # of workers as a function of worker size *x* for the low resource environment. The solid grey line represents the mean age when colonies were relocated from the laboratory into the field.

High-low: Colony age and worker size composition both had significant effects on larval survival in the high-low resource environment. Larval survival decreased with more smaller workers (Fig S4.7d) and at older colony ages (Fig S4.7c). Since worker size was the lowest at older colony ages and highest at younger colony ages (Fig S4.7b), this suggests that colony age and WSC have confounding effects.


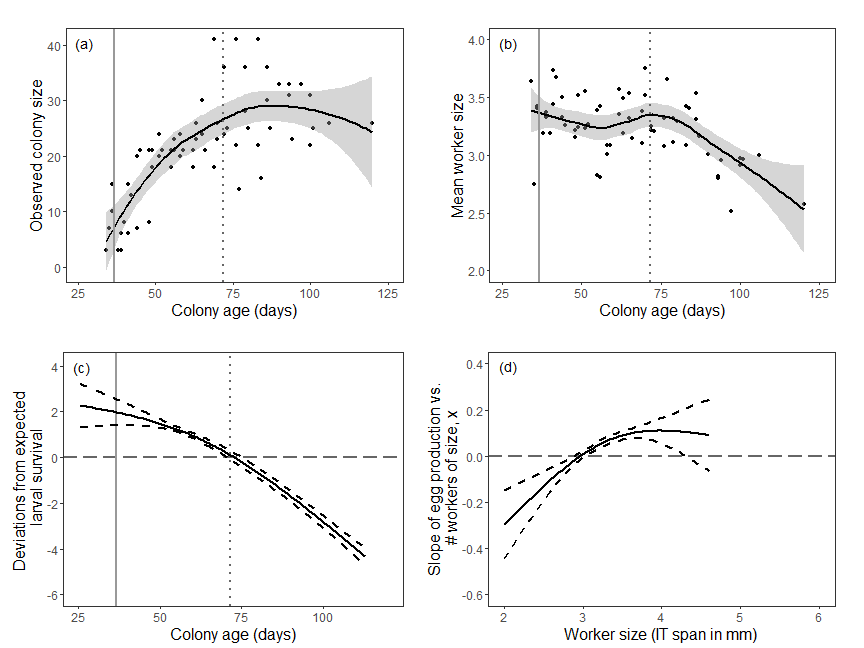


**Figure S4.7.** The (a) observed colony size (i.e. number of workers) and (b) mean worker size plotted against colony age in the high-low resource environment. Smooth components of generalized linear models evaluating the (c) deviations from expected larval survival as a function of colony age and (d) the slope of larval survival vs. # of workers as a function of worker size *x* for the high-low resource environment. The solid grey line represents the mean age when colonies were relocated from the laboratory into the field, and the dotted vertical line represents the end of the early season pulse for the high-low resource environment.

High: Colony age and worker size composition both had significant effects on larval survival in the high resource environment. Larval survival decreased with more workers (Fig S4.8d). Larval survival was also the lowest at 80 days old (Fig S4.8a) when colony size was the highest (Fig. S4.8a). Therefore, colony age and WSC have confounding effects.


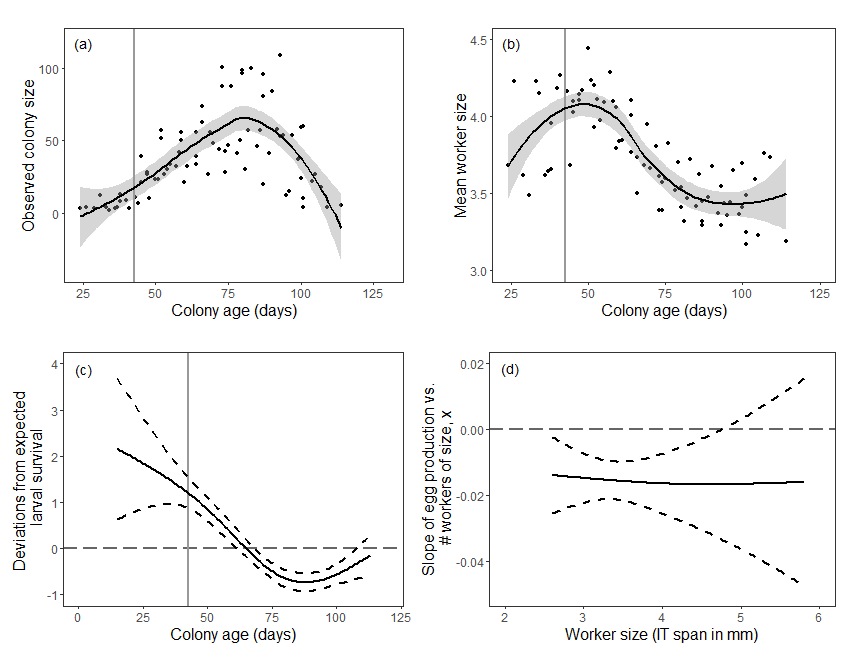


**Figure S4.8.** The (a) observed colony size (i.e. number of workers) and (b) mean worker size plotted against colony age in the high resource environment. Smooth components of generalized linear models evaluating the (c) deviations from expected larval survival probability as a function of colony age and (d) the slope of larval survival vs. # of workers as a function of worker size *x* for the high resource environment. The solid grey line represents the mean age when colonies were relocated from the laboratory into the field.

1. *Mean callow size*

Low: Colony age and worker size composition both had significant effects on mean callow size in the low resource environment. More smaller workers decreased mean callow size (Fig. S4.9d), and worker size seems to be slightly lower at older colony ages (Fig. S4.9b) when colony size was the highest (Fig. S4.9a) and mean callow size was the lowest (Fig. S4.9c). Therefore, these smooth terms are likely to be confounding.


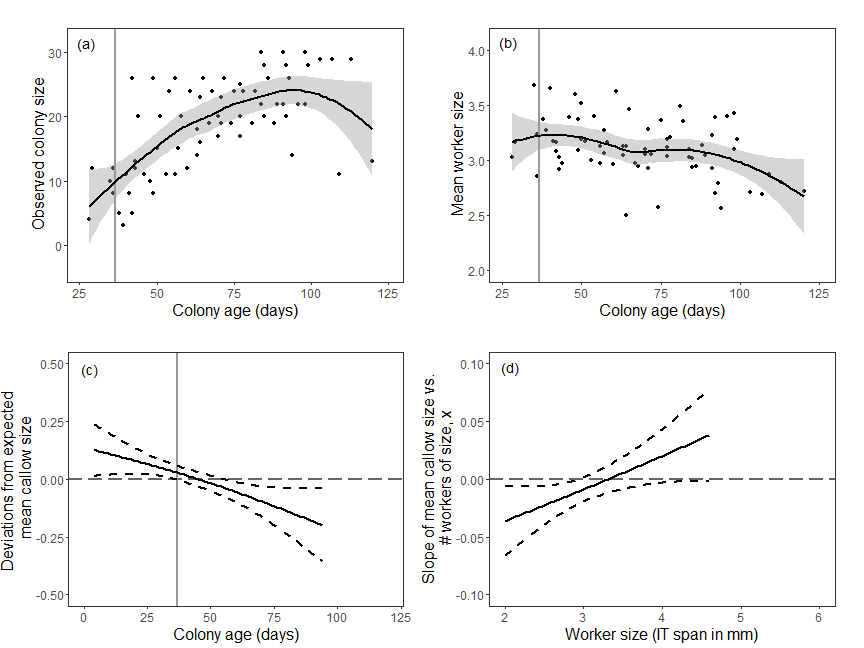


**Figure S4.9.** The (a) observed colony size (i.e. number of workers) and (b) mean worker size plotted against colony age in the low resource environment. Smooth components of generalized linear models evaluating the (c) deviations from expected mean callow size as a function of colony age and (d) the slope of mean callow size vs. # of workers as a function of worker size *x* for the low resource environment. The solid grey line represents the mean age when colonies were relocated from the laboratory into the field.

High-low: Colony age and worker size composition both had significant effects on mean callow size in the high-low resource environment. More smaller workers decreased mean callow size (Fig. S4.10d), and worker size was the lowest at older colony ages (Fig. S4.10b) when colony size was the highest (Fig. S4.10a) and mean callow size was the lowest (Fig. S4.10c). More larger workers increased mean callow size (Fig. S4.10d), and worker size was the highest when the colony was 75 days old or less (Fig. S4.10b) when colony size was the lowest (Fig. S4.10a) and mean callow size was the highest (Fig. S4.10c). Therefore, colony age and WSC are likely to be confounding.


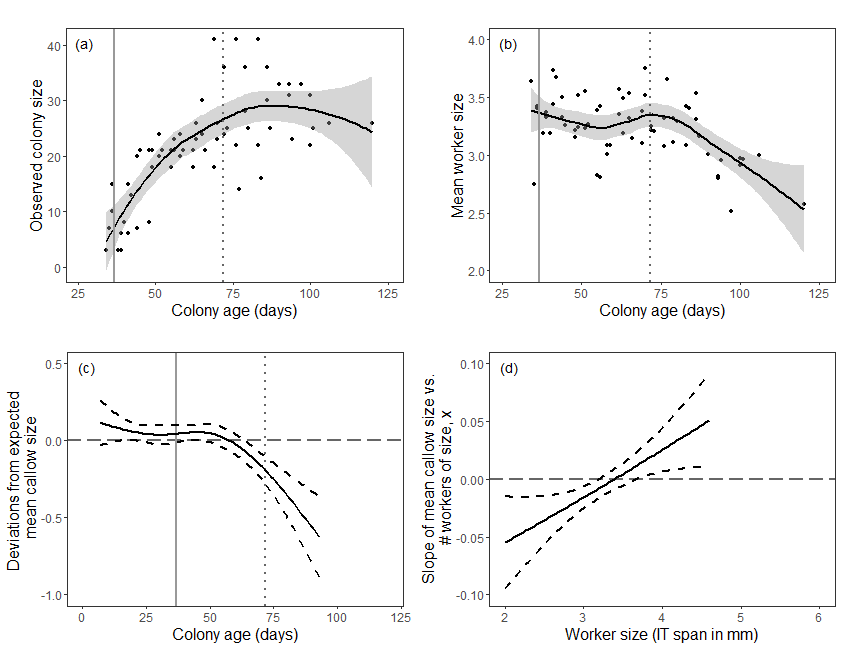


**Figure S4.10.** The (a) observed colony size (i.e. number of workers) and (b) mean worker size plotted against colony age in the high-low resource environment. Smooth components of generalized linear models evaluating the (c) deviations from expected mean callow size as a function of colony age and (d) the slope of mean callow size vs. # of workers as a function of worker size *x* for the high resource environment. The solid grey line represents the mean age when colonies were relocated from the laboratory into the field, and the dotted vertical line represents the end of the early season pulse for the high-low resource environment.

High: Colony age and worker size composition both had significant effects on mean callow size in the high resource environment. Mean callow size with more workers (Fig S4.11d), and colony size was the highest around 80 days (Fig. S4.11a) when mean callow size was the lowest (Fig. S4.11c). Therefore, these smooth terms are likely to be confounded.

**
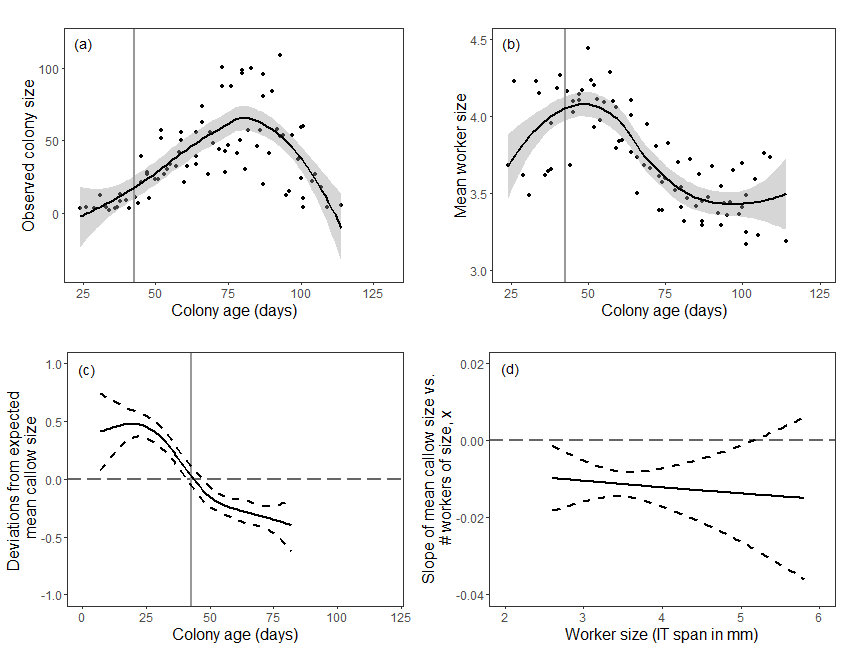
**

**Figure S4.11.** The (a) observed colony size (i.e. number of workers) and (b) mean worker size plotted against colony age in the high resource environment. Smooth components of generalized linear models evaluating the (c) deviations from expected mean callow size as a function of colony age and (d) the slope of mean callow size vs. # of workers as a function of worker size *x* for the high resource environment. The solid grey line represents the mean age when colonies were relocated from the laboratory into the field.

1. *Coefficient of variation in callow size*

Low: Neither colony age nor worker size contribution had significant effects on CV in callow size in the low resource environment. Therefore, we did not need to evaluate confounding effects for this vital rate.

High-low: Colony age and worker size composition both had significant effects on the CV in callow size in the high-low resource environment. More smaller workers increased the CV in callow size (Fig. S4.12d), and worker size is lowest at older colony ages (Fig. S4.12b) when colony size is the lowest (Fig. S4.12a) and CV in callow size is the highest (Fig. S4.12c). Therefore, these smooth terms are unlikely to be confounding.


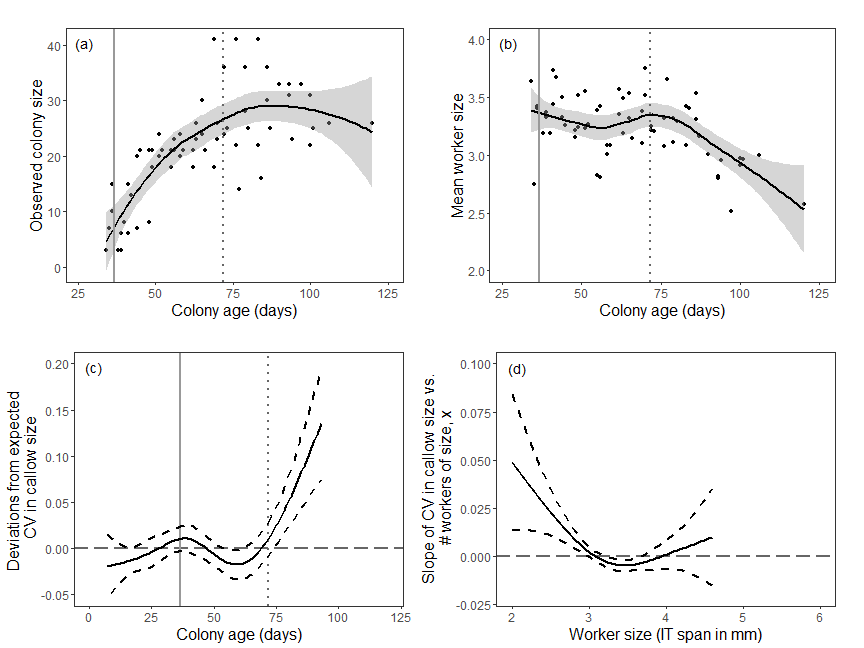


**Figure S4.12.** The (a) observed colony size (i.e. number of workers) and (b) mean worker size plotted against colony age in the high-low resource environment. Smooth components of generalized linear models evaluating the (c) deviations from expected CV in callow size as a function of colony age and (d) the slope of the CV in callow size vs. # of workers as a function of worker size *x* for the high-low resource environment. The solid grey line represents the mean age when colonies were relocated from the laboratory into the field, and the dotted vertical line represents the end of the early season pulse for the high-low resource environment.

High: Neither colony age nor worker size contribution had significant effects on CV in callow size in the high resource environment. Therefore, we did not need to evaluate confounding effects for this vital rate.
